# Neutrophils dominate the immune cell composition in non-small cell lung cancer

**DOI:** 10.1038/ncomms14381

**Published:** 2017-02-01

**Authors:** Julia Kargl, Stephanie E. Busch, Grace H. Y. Yang, Kyoung-Hee Kim, Mark L. Hanke, Heather E. Metz, Jesse J. Hubbard, Sylvia M. Lee, David K. Madtes, Martin W. McIntosh, A. McGarry Houghton

**Affiliations:** 1 Clinical Research Division, Seattle, Washington 98109, USA; 2 Institute of Experimental and Clinical Pharmacology, Medical University of Graz, Universitaetsplatz 4, Graz 8010, Austria; 3 Division of Pulmonary and Critical Care Medicine, University of Washington, Campus Box 356522, Seattle, Washington 98195-6522, USA; 4 Public Health Sciences Division, Fred Hutchinson Cancer Research Center, 1100 Fairview Avenue N, Seattle, Washington 98109, USA; 5 Human Biology Division, Seattle, Washington 98109, USA

## Abstract

The response rate to immune checkpoint inhibitor therapy for non-small-cell lung cancer (NSCLC) is just 20%. To improve this figure, several early phase clinical trials combining novel immunotherapeutics with immune checkpoint blockade have been initiated. Unfortunately, these trials have been designed without a strong foundational knowledge of the immune landscape present in NSCLC. Here, we use a flow cytometry panel capable of measuring 51 immune cell populations to comprehensively identify the immune cell composition and function in NSCLC. The results show that the immune cell composition is fundamentally different in lung adenocarcinoma as compared with lung squamous cell carcinoma, and that neutrophils are the most prevalent immune cell type. Using T-cell receptor-β sequencing and tumour reactivity assays, we predict that tumour reactive T cells are frequently present in NSCLC. These results should help to guide the design of clinical trials and the direction of future research in this area.

## Abstract (web summary)

Tumour immune evasion can involve multiple strategies. Here, the authors characterize the immune populations from clinical specimens of lung cancer in conjunction with TCR-β sequencing and show abundant neutrophils affecting cytotoxic T-cell content and the frequent presence of tumour-specific T-cell clones.

The recent success of immune checkpoint inhibitor (ICI) therapy for non-small cell lung cancer (NSCLC) has galvanized the field. Unfortunately, just ∼20% of NSCLC patients respond to anti-PD1/PDL1 therapy^1^,^2^. ICI therapy likely fails for one of two fundamental reasons: (1) an antigen-driven immune response is not present (that is, it exists in some but not all cases); or (2) an antigen-driven immune response is present, but one or more immune suppressive factors^3^,^4^,^5^,^6^ reside within the tumour microenvironment (TME) that function to derail an otherwise effective immune response.

As is the case with many solid tumour malignancies, NSCLC is a very heterogeneous disease comprised of multiple unique histologic subtypes that harbour distinct molecular signatures^7^. NSCLC is typically subdivided into lung adenocarcinoma (L-ADCA) and lung squamous cell carcinoma (L-SCCA), which account for ∼70% and ∼20% of NSCLC, respectively^8^. Just as the anatomical location and mutational signature of the NSCLC subtypes differ, one would expect that the immune cell composition and function would also differ by NSCLC subtype, if not from case to case. Given the emergence of novel immune-based drugs, a strong foundational knowledge of the immune cell composition and function in NSCLC, and in other solid tumours as well, will likely prove prerequisite to realizing the full potential of such reagents.

In the absence of clear mechanistic evidence to explain ICI treatment failures, numerous early phase clinical trials have been initiated that test additional immune-based therapeutics in conjunction with anti-PD1 therapy^9^. Unfortunately, the field of solid tumour immunotherapy is moving so rapidly that the selection of combinatorial agents has largely been based on theoretical considerations. The malignant component of L-ADCA and L-SCCA has been profiled comprehensively at the molecular level, including the mutational spectra and other molecular features^10^,^11^,^12^. However, a comprehensive resource of immune cell composition and function in NSCLC does not exist. There have been recent attempts to profile the immune cell content of NSCLC and other solid tumour malignancies using transcriptional profiling data^13^,^14^. Since transcriptional signatures have not been conclusively shown to represent actual cellular content, we chose to use flow cytometry to comprehensively profile the immune cell content and function present in NSCLC in attempts to identify the predominant immune cell types present within the TME that could inform therapeutic decision making. In addition, we performed tumour reactivity assays with tumour-infiltrating lymphocyte (TIL) populations on a subset of *N*=10L-ADCA specimens and used this data to predict the frequency with which tumour reactive T cells exist in NSCLC. The results show that tumour-associated T-cell clones are nearly ubiquitous in NSCLC, though their expansion is variable. For all cases, whether or not an antigen-driven immune response is present, neutrophils dominate the immune landscape of NSCLC.

## Results

### Tumour-specific TCR-β sequences are present in NSCLC

**Figure 1:**
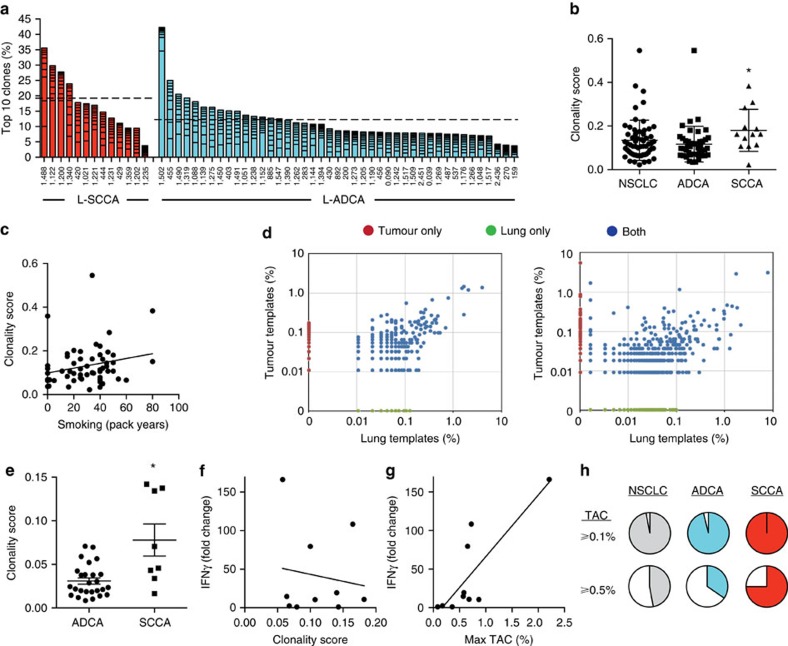
Expansion and specificity of TCR-β repertoire in NSCLC. (**a**) Frequency of TCR-β Top 10 Clones (mean=dotted line, L-SCCA (red) versus L-ADCA (blue), *P=0.0133) were analysed using Adaptive Biotechnologies TCR sequencing platform (*N*=56). Frequency of Top 10 clones is comprised of 10 most expanded clones from each specimen (represented as stacked bar graph). (**b**) Productive clonality score (L-ADCA versus L-SCCA, *P=0.0226, *N*=60) and (**c**) linear correlation of productive clonality score and smoking consumption (pack year) (*P*=0.0821, *R*^2^=0.0540, *N*=58). (**d**) Representative scatter plots for matched lung-tumour specimen with no expanded tumour TAC (left) and expanded TAC (right). TACs were defined as clones only found in tumour specimen (red) but not in matched lung specimen (green). (**e**) Clonality score was calculated for TAC (L-ADCA versus L-SCCA, *P=0.0004, *N*=34). (**f**) Linear correlation of productive clonality score and TIL IFNγ production (*P*=0.6684, *r*^2^=0.0241, *N*=10) and (**g**) linear correlation of maximum (max) TAC and TIL IFNγ production (*P*=0.0035, *r*^2^=0.6357, *N*=10). (**h**) Patients with TAC for NSCLC, L-ADCA and L-SCCA. TACs were defined as clones only present in tumour expanded more than ≥0.1% (top row) and ≥0.5% (bottom row). Student's tests were performed to compare two groups (**a**,**b**,**e**) and Pearson correlation calculations were performed for linear correlations (**c**,**f**,**g**). Each data point represents one patient sample and data are presented as mean±s.e.m., *P<0.05.

Results of checkpoint inhibitor studies support the existence of clonally expanded T cells to tumour-specific antigens^15^. The frequency with which tumour-specific T-cell clones exist and the frequency with which they can expand in NSCLC are unknown. To address these questions, we performed TCR-β sequencing (Adaptive Biotechnologies^16^) on *N*=34 paired lung tumour and non-adjacent lung tissue specimens and an additional *N*=26 tumour specimens for which lung tissue was not available (*N*=4 were rare histological subtypes and not presented in Fig. 1a). Initially, we assessed the top 10 clones (% of total T-cell templates originating from the 10 most frequent sequences) and productive clonality (a measurement of the amount of T-cell clonal expansion within a population of T cells, see Methods for details) present within the tumour specimens. The results were quite variable, with some cases displaying highly expanded clones and others effectively without any expanded populations. Notably, both the top 10 scores (*P*=0.0133, Student's *t*-test) and productive clonality (*P*=0.0226, Student's *t*-test) were significantly higher in the L-SCCA group than in the L-ADCA group (Fig. 1a,b). It has been shown that cigarette smoking drives the high mutational burden in lung cancer^17^, and that these mutated proteins can elicit antigen-driven immune responses^18^. Therefore, we assessed whether cigarette smoking consumption in pack-years (average daily consumption in packs × number of years smoked) influenced TCR-β clonality. As depicted in Fig. 1c, cigarette consumption did not significantly correlate with clonality, likely pointing out that numerous factors can influence T-cell expansion within the lung.

We performed log_10_ scatter plots on all cases for which we had paired TCR-β sequence data to identify sequences that were present in the tumour specimen (at least 0.1% of all T-cell templates) that were not identified within the lung tissue of the same patient. Such sequences were termed tumour-associated clones (TAC) (Fig. 1d). Somewhat surprisingly, TACs are nearly ubiquitous in NSCLC (only one L-ADCA case did not possess such a sequence). However, the ability of a TAC to expand was variable (some cases did not show evidence of an expanded TAC, Fig. 1d, left, whereas others did, Fig. 1d, right), and, similar to measures of clonality and the Top 10 Score, more likely to occur in L-SCCA than in L-ADCA (Fig. 1e).

It is generally assumed that ICI responsive NSCLC patients harbour tumour reactive T-cell clones^15^. Out of the ∼80% of NSCLC patients that do not respond to ICI therapy, the percentage of cases that actually harbour tumour reactive T cells is unknown. To determine whether any of the above measures of TCR repertoire could predict the presence of tumour reactive T cells, we generated tumour-infiltrating lymphocyte (TIL) populations from a cohort of N=10L-ADCA cases for which we also obtained paired TCR-β sequencing data. Detailed methods of TIL culturing are provided in Methods. Expanded TIL populations were tested for tumour reactivity by subjecting the supernatant of TIL:autologous tumour specimen co-culture to IFNγ ELISA to detect IFNγ release. Using TIL and tumour only specimens as controls, *N*=7 cases met the criteria for tumour reactivity (fivefold increase in IFNγ release) whereas the other *N*=3 did not. Next, we assessed whether tumour reactivity as measured by direct co-culture with tumour correlated with either clonality or the presence of a TAC. The maximal TAC value for each case was highly correlated with tumour reactivity (*r*^2^=0.6357, *P*=0.0035, Pearson correlation), whereas clonality was not (Fig. 1f,g). Analysis of the graphical data present in Fig. 1g shows that each case displaying tumour reactivity possessed a TAC>0.5% of all T-cell templates. Using this figure as a cutpoint, we re-analysed the data in pairwise fashion using the Fisher exact test, which revealed a *P* value=0.0083. Lastly, we assessed the frequency with which lung cancers possessed a TAC>0.5% and found that such clones are encountered in nearly half of NSCLC cases, although they are considerably less common in L-ADCA (33%) than in L-SCCA (75%) (Fig. 1h).

### Robust immune response in NSCLC

**Figure 2:**
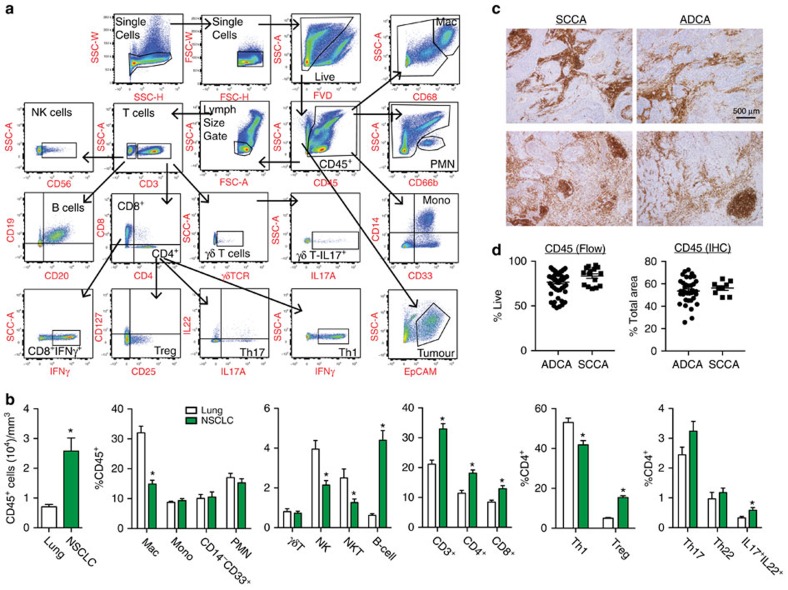
Robust immune cell infiltration in NSCLC. (**a**) Representative polychromatic dot plots demonstrating the gating strategy employed to identify immune cell content in NSCLC. Starting at the top left, initial two gates are to eliminate doublets from the analysis followed by gating on live (FVD) and CD45^+^ cells. For lymphocyte analysis, a size gate was applied followed by CD3 to identify: NK cells (CD3^−^CD56^+^), B cells (CD3^−^CD19^+^CD20^+^), γδT-cells (CD3^+^γδTCR^+^) and CD3^+^CD8^+^ and CD3^+^CD4^+^ T cells. T-cell subsets are displayed including CD8^+^INFγ^+^, Tregs (CD4^+^CD25^+^CD127^lo^), Th17 (CD4^+^IL17A^+^), Th22 (CD4^+^IL22^+^), Th1 (CD4^+^INFγ^+^) and γδTCR^+^IL17A^+^. Myeloid lineage cells were gated on CD45^+^ cells: neutrophils (CD66b^+^), monocytes (CD14^+^CD33^+^) and macrophages (CD68^hi^). Tumour cells were defined as CD45^−^EpCAM^+^. (**b**) Tabulation of immune cell content from non-adjacent lung (white bars) and NSCLC (green bars) flow cytometry data. Data presented as CD45^+^ cells per mm^3^, % CD45^+^, % CD4^+^, or % parent, as indicated. (**c**) Representative CD45 IHC depicting immune cell distribution in L-SCCA and L-ADCA specimens. Scale bar, 500 μm (**d**) Tabulation of CD45 content in L-ADCA and L-SCCA measured by flow cytometry (*N*=73, left) and IHC (*N*=46, right). Student's tests were performed to compare two groups (**b**,**d**). Data are presented as mean±s.e.m., *P<0.05.

To identify the dominant immune suppressive factors present in NSCLC, we comprehensively profiled the immune cell content and function in a prospective cohort of 73 consented subjects undergoing surgical resection of lung cancer for curative intent (Supplementary Table 1). We employed a flow cytometry panel composed of 27 markers (Supplementary Table 2) that can identify 51 unique immune cell types and functional subpopulations using single-cell suspensions generated from lung cancer tissue and non-adjacent lung tissue (as far removed from the tumour lesion as possible, at least 3 cm). The gating strategy is depicted in Fig. 2a, and the details of tissue processing, staining, gating, data analysis and statistical analyses are provided in the Methods, Supplementary Figs 1–3, and Supplementary Tables 3–7. The data were analysed in multiple ways, including % live, % CD45^+^, % parent and matched tumour-normal pairs, while accounting for smoking and other clinical features, all of which produced similar results (Supplementary Tables 3–6).

NSCLC elicited and/or generated a robust immune response in which CD45^+^ immune cells were more prevalent in tumour specimens than were cancer cells, representing ∼75% of the cellular content located within tumours. Tumour lesions contained three times the numbers of immune cells (cells per mm^3^ tissue) than were identified in non-adjacent lung tissue (Fig. 2b). Since tumour digestion protocols may preferentially recover cells that already exist as single cells (that is, immune cells) over aggregated cells (that is, tumour cells), it is possible that the CD45^+^ cell figure enumerated by flow cytometry represented an overestimate. Therefore, we performed CD45 immunohistochemistry (IHC) on all cases for which we could access the tissue blocks. Tabulation of CD45 content using HALO image analysis software (see Methods) demonstrated that >50% of the tumour area in NSCLC is occupied by cells staining positively for CD45, thereby confirming the robust nature of leukocyte content in NSCLC (Fig. 2c,d). Analysis of CD45^+^, CD3^+^ and CD4^+^ subset composition revealed that subgroups of immune cells associated with tumour or normal lung (Supplementary Fig. 4). Compared with normal lung tissue, NSCLC specimens displayed increased composition of 37 distinct immune cell types and subtypes, including for B cells (CD19^+^CD20^+^), T cells (CD3^+^), CD4^+^ cells and CD8^+^ cells. The expansion of B cells is notable as they increased seven-fold compared with lung tissue, the largest fold-change for any immune cell type. The composition of CD4^+^ subsets differed as well, as evidenced by a statistically significant increase in Tregs, defined as CD4^+^CD25^+^CD127^lo^ cells^19^ (Supplementary Fig. 5), and decrease in Th1 cells (CD4^+^IFNγ^+^; Fig. 2b). Detailed analysis of all immune cell types and subtypes is provided in Supplementary Figs 6–8 and Supplementary Tables 3–5).

### Differential CD4^+^ subset composition for L-ADCA vs L-SCCA

**Figure 3:**
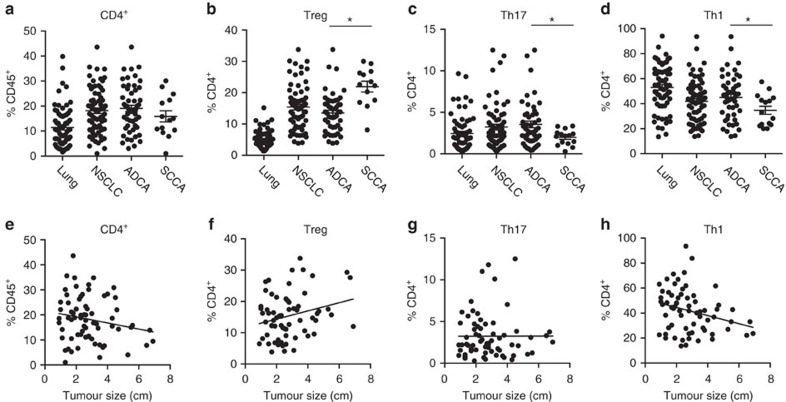
Differential CD4^+^ subset composition for L-ADCA versus L-SCCA. (**a**) CD4^+^ cells displayed as % CD45^+^ cells, (**b**) Treg (L-ADCA versus L-SCCA, *P<0.0001, *N*=63), (**c**) Th17 (L-ADCA versus L-SCCA, *P=0.0353, *N*=63), and (**d**) Th1 cells (L-ADCA versus L-SCCA, *P=0.0471, *N*=63) shown as per cent of CD4^+^ cells. Linear correlations of tumour size (cm) and (**e**) CD4^+^ cells (*P*=0.1113, *R*^2^=0.0369, *N*=53), (**f**) Treg (*P=0.0402, R^2^=0.0642, *N*=50), (**g**) Th17 (*P*=0.9848, *R*^2^=0.0000, *N*=48) and (**h**) Th1 cells (*P=0.0286, *R*^2^=0.0727, *N*=48) in L-ADCA specimen. Student's tests were performed to compare two groups (**a**–**d**) and Pearson correlation calculations were performed for linear correlations (**e**–**h**). Each dot represents an independent data point as determined by flow cytometry. Data are presented as mean±s.e.m., *P<0.05.

Since Tregs, Th17 and Th1 cells impact tumour growth in disparate ways^20^, we employed an intracellular stimulation protocol to directly measure CD4^+^ subset composition. Whereas total CD4^+^ content was unable to distinguish between L-ADCA and L-SCCA, the relative composition of the CD4^+^ subsets was able to distinguish between the two. Specifically, L-SCCA tumours contained twice as many Tregs as L-ADCA specimens, with concomitant reductions in Th17 and Th1 lymphocytes (Fig. 3). Another key feature of these data is that the immune cell composition is more heterogeneous in L-ADCA than L-SCCA (Supplementary Fig. 9), as evidenced by the tight clustering of immune cell composition values from L-SCCA specimens depicted in Fig. 3b–d. Once again, the CD4^+^ subsets represent the group that best illustrates the differences in heterogeneity between L-SCCA and L-ADCA (Fig. 3, Supplementary Fig. 9 and Supplementary Table 3–5). Importantly, Th1 cells were the only cell type in our study that inversely correlated with tumour size, consistent with their well-accepted pro-host role in cancer (Fig. 3e–h). In contrast, Tregs positively correlated with tumour size (*P*=0.042, Pearson correlation).

### CD4^+^ and CD8^+^ function segregate by NSCLC subtype

**Figure 4:**
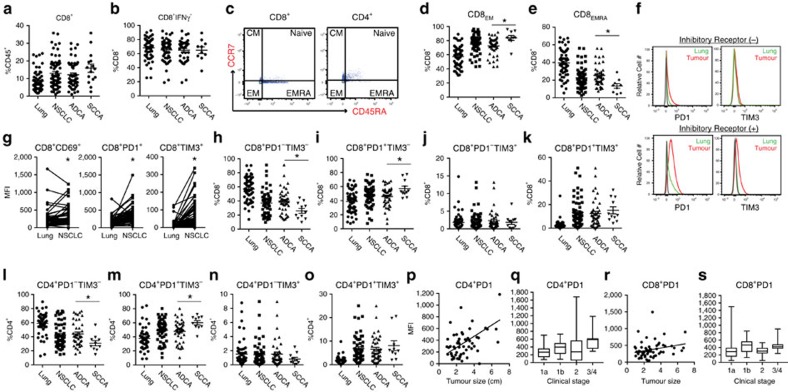
CD4^+^ and CD8^+^ function segregate by NSCLC subtype. (**a**) CD8^+^ cells shown as % CD45^+^ and (**b**) CD8^+^IFNγ^+^ shown as per cent of CD8^+^. (**c**) Representative dot plots showing CD8^+^ and CD4^+^ cell subtypes (CCR7^−^CD45RA^−^ effector memory, CCR7^−^CD45RA^+^ effector memory RA, CCR7^+^CD45RA^−^ central memory, CCR7^+^CD45RA^+^ naïve). Statistical analyses of (**d**) CD8^+^_EM_ (L-ADCA versus L-SCCA, *P=0.0123, *N*=54) and (**e**) CD8^+^_EMRA_ (L-ADCA versus L-SCCA, *P=0.0064, *N*=54). (**f**) Representative histograms of low (top) and high expression of inhibitory receptors (bottom) on CD8^+^ cells determined by expression (MFI) of PD1 (left) and TIM3 (right). Matched normal lung-tumour pairs for (**g**) CD8^+^CD69^+^ (left, *P=0.0003, *N*=55). CD8^+^PD1^+^ (middle, *P<0.0001, *N*=56) and CD8^+^TIM3^+^ (right, *P<0.0001, *N*=56) content. (**h**) CD8^+^PD1^−^TIM3^−^ (*P=0.0156, *N*=53), (**i**) CD8^+^PD1^+^TIM3^−^ (*P=0.0188, *N*=53), (**j**) CD8^+^PD1^−^TIM3^+^, (**k**) CD8^+^PD1^+^TIM3^+^ (**l**) CD4^+^PD1^−^TIM3^−^ (*P=0.027, *N*=51), (**m**) CD4^+^PD1^+^TIM3^−^ (*P=0.0195, *N*=51), (**n**) CD4^+^PD1^−^TIM3^+^ and (**o**) CD4^+^PD1^+^TIM3^+^ flow cytometry data displayed as %CD8 or %CD4 for L-ADCA and L-SCCA. Associations of CD4^+^PD1 and (**p**) tumor size (linear correlation, *P*=0.0001, *R*^2^=0.2386, *N*=43), (**q**) clinical stage (ANOVA, stage 1a versus 3/4, *P*=0.0277, *N*=43) and associations of CD8^+^PD1 and (**r**) tumour size (linear correlation, *P*=0.0598, *R*^2^=0.0629, *N*=43) and (**s**) clinical stage (ANOVA, stage 1a versus 3/4, *P*=0.1798, *N*=43). Student's tests were performed to compare two groups (all except, **c**,**f**,**p**–**s**), Pearson correlation calculations were performed for linear correlations (**p**,**r**) and one-way ANOVA analysis was performed for multiple comparisons (**q**,**s**). Each dot represents an independent data point as determined by flow cytometry. Data are presented as mean±s.e.m. *P<0.05. ANOVA, analysis of variance.

Tumour-infiltrating CD8^+^ cells have been positively correlated with patient outcomes for several solid tumour types^21^. In our cohort, CD8^+^ cell composition was higher in NSCLC specimens than in non-adjacent lung tissue (Fig. 4a), though total CD8^+^ cellular content did not correlate with tumour size (Supplementary Table 6). There was a trend toward increased CD8^+^ content in L-SCCA when compared with L-ADCA, though this did not reach statistical significance. In contrast to the clear segregation of CD4^+^ subsets, CD8^+^ cellular content was rather heterogeneous in L-ADCA and L-SCCA alike. Furthermore, IFNγ production by CD8^+^ cells was equivalent in lung tumour and lung tissue and was not impacted by NSCLC histological subtype (Fig. 4b). Since the measurement of IFNγ production involved *ex vivo* stimulation, these results may not accurately reflect the *in vivo* behaviour of CD8^+^ T cells in this regard.

Functional analyses of CD4^+^ and CD8^+^ populations revealed the presence of cellular subsets unique to NSCLC histological subtypes. For example, effector/memory status (as determined by CCR7 and CD45RA staining^22^) showed a decrease in CD8_EMRA_ content (CD8^+^CCR7^−^CD45RA^+^) in tumour tissue compared with lung tissue with a reciprocal increase in CD8_EM_ content (CD8^+^CCR7^−^CD45RA^−^) in tumour tissue compared with lung tissue (Fig. 4c–e). Notably, CD8_EMRA_ cells were significantly decreased in L-SCCA compared with L-ADCA. Measures of effector memory status in CD4^+^ cells failed to segregate CD4^+^ subpopulations by cancer type.

Tumour-associated CD8^+^ cells uniformly expressed the activation marker CD69 (ref. 23), but displayed variable levels of the inhibitory receptors PD1 and T-cell immunoglobulin domain and mucin domain 3 (TIM3)^24^. Both markers were significantly higher in NSCLC specimens compared with matched lung tissue controls, (Fig. 4f,g). CD8^+^PD1^+^ content was significantly elevated in L-SCCA as compared with L-ADCA (Fig. 4h–k). Similar to the findings with CD8^+^ cells, CD4^+^PD1^+^ content was significantly higher in NSCLC specimens than in matched lung tissue, and in L-SCCA compared with L-ADCA (Fig. 4l–o). Furthermore, PD1 expression on CD4^+^ cells was associated with both increased tumour size and advanced clinical stage (Fig. 4p,q). In contrast, CD8^+^PD1 expressing cells did not correlate with size or stage, though there was a trend towards increased size (*P*=0.059, Pearson correlation; Fig. 4r,s).

We also assessed the surface expression of PDL1 by cellular subtype. Multiple different cell types residing within the lung TME express PDL1, including macrophages, monocytes, neutrophils and epithelial cell adhesion molecule (EpCAM)^+^ tumour cells (Supplementary Fig. 10). As has been reported by IHC, the expression of PDL1 is quite diverse, with many cases expressing little to no PDL1. Significantly greater PDL1 expression was measured on macrophages, monocytes, neutrophils, the CD45^−^ population, and EpCAM^+^ cells in tumour when compared with lung tissue but no differences were observed between NSCLC subtypes (Supplementary Fig. 10). Notably, macrophages and monocytes expressed significantly greater PDL1 than did any other cellular population, including the Epcam^+^ population (Supplementary Fig. 10g).

### Neutrophils are the most abundant immune cell type in NSCLC

**Figure 5:**
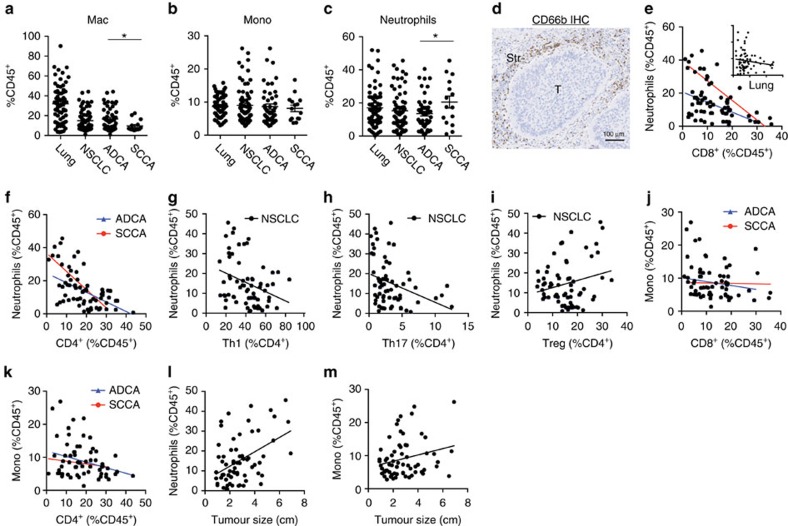
Neutrophils are the dominant immune cell type in NSCLC. (**a**) Macrophages (L-ADCA versus L-SCCA, *P=0.0198, *N*=64), (**b**) monocytes and (**c**) neutrophils (L-ADCA versus L-SCCA, *P=0.0328, *N*=65) shown as % CD45^+^. (**d**) Representative CD66b IHC depicting neutrophil distribution in stromal (Str) areas in NSCLC. Scale bar, 100 μm. Linear correlation between (**e**) neutrophils and CD8^+^ (L-ADCA (blue): *P=0.0013, *R*^2^=0.2000; L-SCCA (red): *P=0.0008, R^2^=0.5916, *N*=64, lung (inset): *P*=0.2285, R^2^=0.02189, *N*=68), (**f**) neutrophils and CD4^+^ (L-ADCA (blue): *P<0.0001, *R*^2^=0.3174; L-SCCA (red): *P=0.0077, *R*^2^=0.4592, *N*=63), (**g**) neutrophils and Th1 (NSCLC: *P=0.0079, R^2^=0.1050, *N*=66), (**h**) neutrophils and Th17 (NSCLC: *P=0.0068, R^2^=0.1088, *N*=66), (**i**) neutrophils and Tregs (NSCLC: *P*=0.0623, *R*^2^=0.0549, *N*=64), (**j**) monocytes and CD8^+^ (L-ADCA (blue): *P*=0.2403, *R*^2^=0.0327; L-SCCA (red): *P*=0.9069, *R*^2^=0.0011, *N*=59) and (**k**) monocytes and CD4^+^ (L-ADCA (blue): *P=0.0172, R^2^=0.1279; L-SCCA (red): *P*=0.5558, *R*^2^=0.02970, *N*=58). Linear correlations of tumour size and (**l**) neutrophils (*P=0.0001, *R*^2^=0.2034, *N*=67) and (**m**) monocytes (*P=0.0498, *R*^2^=0.05878, *N*=66) for NSCLC specimen measured by flow cytometry. Student's tests were performed to compare two groups (**a**–**c**) and Pearson correlation calculations were performed for linear correlations (**e**–**m**). All data determined by flow cytometry and each dot represents independent data point. Data are presented as mean±s.e.m., *P<0.05 unless otherwise indicated.

Cells of myeloid lineage are abundant within the lung TME and account for ∼50% of tumour-infiltrating CD45^+^ cells (Fig. 5). The percentage of macrophages is statistically reduced in NSCLC specimens versus lung tissue, which is likely a function of the reciprocal nature of presenting data as CD45%, as opposed to absolute cellular content (not shown). However, such analytical concepts do not explain the significant reduction in macrophage content in L-SCCA compared with L-ADCA (Fig. 5a). Monocyte content is similar between lung tissue and NSCLC tissue, and between L-SCCA and L-ADCA (Fig. 5b). Neutrophils were the most abundant immune cell type identified in NSCLC specimens, accounting for nearly 20% of all CD45^+^ cells. Although their content was similar between lung tissue and tumour tissue, L-SCCAs contained more neutrophils than L-ADCAs (Fig. 5c). The neutrophils (using CD66b IHC) were predominantly located within the tumour stroma in the limited number of cases (*N*=12) that we analysed in this manner (Fig. 5d).

Since cells of both the neutrophil and monocyte lineages have been demonstrated to possess lymphocyte suppressive capabilities^25^, we performed linear regressions to identify which myeloid cells were inversely associated with lymphocytes. This analysis revealed a strong negative correlation between neutrophil and CD8^+^ cellular content in both L-ADCA and L-SCCA (Fig. 5e). Notably, this association did not exist in non-adjacent lung tissue (Fig. 5e, inset), strongly suggesting that this is a tumour-specific phenomenon. We also found that neutrophil content negatively predicted the content of CD4^+^ lymphocytes as a whole, and specifically of Th1 and Th17 cellular subpopulations (Fig. 5f–h). Of the CD4^+^ subsets, only Treg content was not impacted by the presence of neutrophils (Fig. 5i). In contrast to the findings with respect to neutrophils, we were unable to associate monocyte (CD14^+^CD33^+^) content with CD8^+^ or CD4^+^ content, with the exception that monocytes negatively correlated with CD4^+^ cells specifically in L-ADCA specimens (Fig. 5j,k).

The cell surface markers employed in our primary cohort lacked the ability to distinguish monocytes and neutrophils from their myeloid-derived suppressor cell (MDSC) counterparts. Therefore, we processed a supplementary cohort of *N*=8 NSCLC specimens (Supplementary Fig. 11), in which we assessed the MDSC lineages in more detail using recently published guidelines to choose MDSC markers^26^. Consistent with other studies, most (∼60%) of the monocytes in the blood of NSCLC patients were HLA-DR^lo^ (Supplementary Fig. 11a,c), a population typically referred to as monocytic-MDSC (M-MDSC)^27^. Surprisingly, HLA-DR^lo^ cells represented a minority (∼15%) of the CD14^+^ monocyte population within the tumour specimen itself, with the majority of monocyte lineage cells displaying surface markers consistent with traditional monocytes (CD14^+^CD33^+^HLA-DR^hi^; Supplementary Fig. 11a,c). Thus, while it is clear that monocytes displaying M-MDSC surface markers are abundant in the blood of NSCLC patients, it appears that these cells are less common within the TME. However, monocyte content positively correlated with tumour size and PDL1 expressing monocytes were one of a few cellular subsets to associate with advanced clinical stage (Supplementary Table 7), highlighting important contributions of this cell type within the TME.

Cell surface markers capable of distinguishing neutrophils from PMN-MDSC do not currently exist (Supplementary Fig. 11)^26^. Interestingly, we identified a small population of CD66b^+^HLA-DR^+^ cells in the TME, which likely represents a recently reported subset of neutrophils that can function as antigen presenting cells^28^. Consistent with a recent report, we found that neutrophils from NSCLC patients localized to the low-density monocyte fraction upon sucrose density gradient centrifugation (Supplementary Fig. 11f), whereas the neutrophils from healthy volunteers were located within the high-density fraction, as expected^29^. On the basis of these results, and the lack of functional data, we have simply termed these cells ‘neutrophils' in our study, with the understanding that some of these cells are likely PMN-MDSC, while others are likely neutrophils.

We also identified that ∼10% of all CD45^+^ immune cells found in NSCLC specimens displayed a cellular phenotype of CD45^+^CD14^−^CD68^−^CD66b^−^CD33^+^, labelled here as CD14^−^CD33^+^ for short (Fig. 2b and Supplementary Fig. 12). We suspect that these cells may represent the recently described ‘early' MDSC cellular subset, but lack all of the lineage markers to make that claim^26^,^30^. Nonetheless, since these cells represented a substantial proportion of the immune cells found in NSCLC specimens, we interrogated their ability to differentiate between NSCLC histologic subtypes, correlate with other cellular populations and/or tumour size, but found no such associations (Supplementary Fig. 12).

### TCR clonality is associated with CD8 immunity

**Figure 6:**
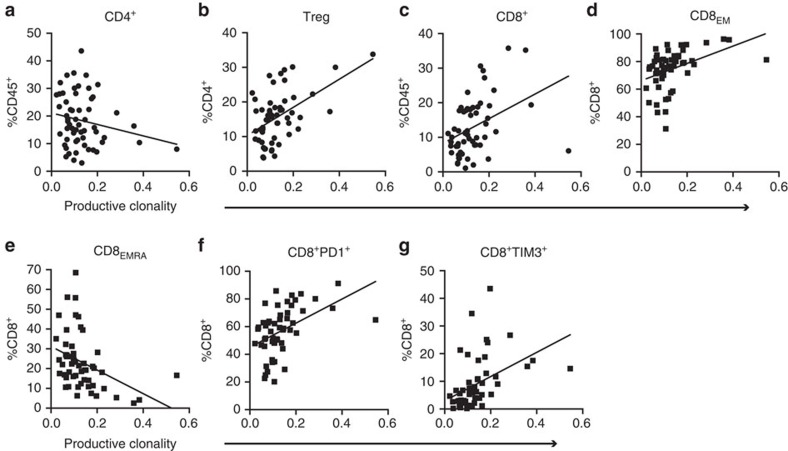
TCR clonality is associated with CD8 immunity. Linear correlation for NSCLC specimens between productive clonality and (**a**) CD4^+^ (*P*=0.1300, *R*^2^=0.0405, *N*=58), (**b**) Treg (*P<0.0001, *R*^2^=0.2641, *N*=57), (**c**) CD8^+^ (*P=0.0021, *R*^2^=0.1567, *N*=58), (**d**) CD8^+^_EM_ (*P=0.0022, *R*^2^=0.1657, *N*=54), (**e**) CD8^+^_EMRA_ (*P=0.0036, *R*^2^=0.1520, *N*=54), (**f**) CD8^+^PD1^+^ (*P=0.0004, *R*^2^=0.2182, *N*=53) and (**g**) CD8^+^TIM3^+^ (*P=0.0011, *R*^2^=0.1910, *N*=53). Each dot represents an independent data point as determined by flow cytometry. Pearson correlation calculations were performed for linear correlations (**a**–**g**). Data are presented as mean±s.e.m., *P<0.05.

To identify which cellular populations were expanded or contracted as a function of TCR-β repertoire, we assessed measurements of TCR-β clonality in the context of immune cell composition for the same case. Initially, we queried whether a relationship between TCR clonality and T-cell content existed. Not surprisingly, we identified a strong correlation (*r*^2^=0.73, *P*<0.0001, Pearson correlation) when correlating TCR clonality with a composite of the dominant T-cell populations (CD4^+^, CD8^+^ and Treg) in our study (not shown). Of these, clonal expansion, when observed, is driven by CD8^+^ cells (Fig. 6a–c), as the CD4^+^ population as a whole does not correlate with clonality. Therefore, we chose to assess the relationship between CD8^+^ lymphocyte subpopulations and clonality, though the strength of these statistical associations was considerably weaker. Measurements of clonality correlated positively with effector memory CD8^+^ cells (CD8^+^CCR7^−^CD45RA^−^) but negatively with effector memory RA populations (CD8^+^CCR7^−^CD45RA^+^) (Fig. 6d,e). Both the PD1 and TIM3 expressing CD8 populations were correlated with clonality, consistent with the concept that these inhibitory receptors are upregulated in response to chronic antigen exposure (Fig. 6f,g).

## Discussion

The malignant portion of NSCLC and many other solid tumour types have been comprehensively profiled at the molecular level, including mutational spectra^10^,^11^,^12^. Recently, similar approaches have been used to profile the immune landscape present in cancer using transcriptional profiling and IHC^14^. Although informative, transcriptional signatures have not been clearly demonstrated to infer immune cell content, nor are they capable of delineating unique immune cell subtypes that require multiple markers to identify. Alternatively, IHC provides critical information regarding the spatial relationships between leukocytes and cancer cells, but only captures a small area of tumour and might not reliably represent tumour heterogeneity.

We chose to use flow cytometry because of its ability to directly and quantitatively measure the immune cell content of a large number of distinct cell types on the same specimen while also obtaining functional information on their behaviour (for example, PD1 expression by CD8^+^ cells). An additional feature of the current study is the inclusion of immune cell compositional data from non-adjacent (normal) lung tissue. For example, the finding that B cells represent 4.4% of the CD45^+^ cells in NSCLC has a completely different connotation with the perspective that B cells represent just 0.6% of immune cell content in lung tissue. The inclusion of this data provides a better understanding of which immune cell populations are unique to the TME.

We obtained each specimen within 2 h of resection, generated single cell suspensions immediately, and performed the flow cytometry studies thereafter, on the same day. Using this protocol, we were able to routinely obtain high levels of cellular viability, and utilized a live/dead marker to ensure the integrity of the data. To our knowledge, the current study represents the largest and most comprehensive flow cytometric analysis of immune cell composition and function for any solid tumour type. One potential limitation of our study is that the required methodology mandates that specimens be collected prospectively, such that hard outcomes data are not immediately available and validation cohorts do not exist. Although we believe that the cellular composition of NSCLC represents important data in the absence of outcomes, we utilized both tumour size and clinical stage as prognostic surrogates. Notably, only five cell types correlated with clinical stage, and these were all PD1 and PDL1 expressing subtypes (Supplementary Table 7). In particular, PD1 expressing CD4^+^ T cells were the only subset to associate with both tumour size and clinical stage in our cohort, which points to a potentially important role for this poorly understood cellular subtype. Nevertheless, future independent validation of the major cellular phenotypes in NSCLC, and inclusion of patients having received prior therapies, is an ongoing effort at our institution.

The purpose of this study was to provide investigators with a clear depiction of the immune landscape present in NSCLC to guide future research endeavours in this area and the design of rapidly emerging clinical trials employing immune-based therapeutics. To that end, we were able to identify unique immune signatures by NSCLC histological subtype, highlighting the heterogeneity inherent in immune responses to cancer. Although immune cell composition clusters relatively well in L-SCCA, it is highly heterogeneous in L-ADCA. One potential explanation of these results is that cigarette smoke consumption and subsequent mutational burden is more variable in L-ADCA than in L-SCCA. In addition, the presence of key driver mutations in L-ADCA (for example, *KRAS*, *EGFR* and so on) may generate unique immune responses that would define further novel subgroups. Unfortunately, since the cohort studied here underwent surgical resection for curative intent, mutational analysis was not clinically indicated.

Recent studies suggest that response to ICIs is associated with the development of clonal expansion within a T-cell population that likely identifies a tumour-specific mutation functioning as a neo-antigen^15^,^18^. Although these studies may explain why certain patients respond to such therapies, they do not provide data with which to generate alternative therapeutic strategies for the 80% of NSCLC patients that initially fail ICI therapy. Here, we show that immune suppressive factors are nearly ubiquitous in NSCLC and that they differ by NSCLC subtype, if not from case to case. Therefore, to achieve optimal treatment benefit from immune-based therapies, it is likely that targeting the other dominant immune suppressive factors will be required. Our data implicates Tregs and neutrophils as potential immune suppressive factors in NSCLC. While there are a number of clinical trials underway that combine anti-PD1 antibodies with agents that should theoretically deplete Tregs, there are effectively no clinical investigations focused on the neutrophil population.

MDSCs have emerged as important, and potentially therapeutically relevant, immune suppressive factors within the TME^25^. Debate persists as to which MDSC subset is the dominant lymphocyte suppressive entity in human cancers *in vivo*, and if the suppressive properties displayed by peripheral blood MDSCs are maintained by their tumour-infiltrating counterparts^31^. Furthermore, much of the MDSC literature is based upon *ex vivo* co-culture experiments, which may or may not accurately represent the interactions between these cells as they exist *in vivo*. Therefore, we chose to identify the associations between lymphocytes and myeloid lineage cells as exists *in vivo*, which should assist in the design of future experiments in this area.

There is substantial confusion regarding the semantics of these cell types, as they share surface marker definitions with other tumour-associated monocytes and granulocytes that do not possess lymphocyte suppressive function^32^. We chose to label CD45^+^CD66b^+^ cells as neutrophils, with the understanding that a subset of these cells would possess lymphocyte suppressive properties while others would be more traditional neutrophils. Surprisingly, the neutrophil (and not the monocyte) population negatively correlated with CD8^+^ content. On the basis of this data, it is tempting to speculate that the neutrophil lineage is the dominant lymphocyte suppressive factor in NSCLC. However, in the absence of functional data on these cells, and the lack of the HLA-DR marker to evaluate M-MDSC within the entire cohort, it is difficult to make such a claim. Nevertheless, there is mounting evidence to support a deleterious role for the neutrophil lineage in NSCLC. Gentles *et al*.^14^ recently identified the neutrophil transcript signature as the strongest predictor of mortality of any immune cell type in a large cohort of NSCLC patients. It is important to note that neutrophils are not very transcriptionally active cells. As such, neutrophils were estimated to represent just ∼2% of immune cells in NSCLC based on transcript abundance, while we show that they are the most common immune cell type at the cellular level, accounting for nearly 20% of all immune cells. Taken together, these studies warrant further interrogation and possible therapeutic manipulation of the neutrophil lineage in NSCLC.

The combined TCR-β sequencing and functional TIL experimental data allowed us to predict that tumour reactive T cells exist in 75% of L-SCCA but in <35% of L-ADCA. Given the technical challenges inherent to TIL generation and autologous tumour co-culture, our TIL cohort was limited to *N*=10 cases. In addition, we did not prove that the tumour reactive clones shared the same sequence as the predominant *in vivo* TAC. As such, independent validation of these findings will be required before TCR-β sequencing could be used clinically to identify the presence of tumour reactive T cells. Although L-SCCAs frequently possess clonal T-cell populations, they uniformly contain an abundance of Tregs and inhibitory receptor (that is, PD1) expressing CD8^+^ cells, with reduced Th1 immunity and a paucity of CD8^+^ effector cells. Given evidence of frequent antigen-driven immune responses, combination of anti-PD1 therapies with novel therapeutics addressing the abundant Treg population may improve response rates. Clinical trials employing such strategies are currently underway. L-ADCA will likely prove much more difficult than L-SCCA to achieve meaningful clinical responses from immune-based therapeutics. L-ADCAs frequently lack expanded TACs and inhibitory receptor expressing CD8^+^ cells, which may indicate that tumour reactive T cells are present less frequently in L-ADCA than L-SCCA subjects. In addition, the presence of key driver mutations may generate polarized immune responses in L-ADCA. However, it may be possible to generate immunogenic tumours by using preconditioning therapies such as radiation^33^ or chemotherapy^34^. For example, oxaliplatin, a so-called ‘immunogenic' chemotherapy, was recently combined with anti-PD1 antibodies to reduce tumour burden in a mutant *Kras* mouse model of lung adenocarcinoma, while anti-PD1 therapy alone was ineffective^35^. Additional investigation into the combination of chemotherapy or targeted therapies with immunotherapies will be required to address the numerous L-ADCA subtypes.

As with NSCLC, most solid tumour malignancies possess more than one major histological subtype that likely generate disparate immune responses. The heterogeneity in the immune cell response to NSCLC highlights the need to develop novel immune diagnostics that could guide both the initial choice of immunotherapy and to devise a secondary strategy to address treatment failures. With the rapidly expanding arsenal of immune-based therapeutics for cancer therapy, utilizing an immune diagnostic test prospectively to target the dominant immune suppressive factors within a given tumour may improve response rates while ushering in the age of personalized immune-based therapies for cancer patients.

## Methods

### Study design

This study was performed on NSCLC tissue and non-adjacent lung tissue (as far removed from the malignant lesion as possible, at least 3 cm) from the same patient obtained from consented subjects at the Fred Hutchinson Cancer Research Center (FHCRC)/University of Washington Hospital/ Northwest BioTrust, under an active IRB protocol. The specimens were obtained prospectively over a 24-month study period and were assayed if there was sufficient material to perform lymphocyte, myeloid and intracellular flow cytometry panels (*N*=73). Additional functional studies were performed on a subset of specimens for which additional tissue was available. These studies include TCR-β sequencing (*N*=60) and CD45 IHC (*N*=46). The primary analyses were to identify unique TCR-β sequences that were present in tumour tissue but not non-adjacent lung tissue, to identify differences in immune cell composition between lung tissue and NSCLC tissue, and between L-ADCA and L-SCCA. Secondary analyses included associations of specific immune cell types with tumour size, and associations between two different immune cell types. Corresponding clinicopathological data was maintained in a highly annotated database to allow for appropriate analyses of co-variates (Supplementary Table 1).

Two additional supplementary cohorts were used in this study. Supplementary cohort #1 consisted of *N*=10L-ADCA cases that were used to generate TIL populations to assess the presence of tumour reactive T-cell populations. Paired TCR-β sequencing data was also obtained for this subgroup. Supplementary cohort #2 consisted of *N*=8 NSCLC patients and *N*=6 healthy volunteer blood donors. The specimens derived from this group were subjected to a detailed MDSC panel.

### Study approval

This study was approved by the Fred Hutchinson Cancer Research Center Institutional Review Board (IRB) and was assigned file no. 6663. All patients signed an informed consent.

### Pathology

In addition to routine H&E staining, each case was subjected to p63, cytokeratin 5 (CK5), Napsin A and TTF-1 IHC to assist in classifying the histologic subtype^36^. Each case was assigned one of the following histological subtypes: ADCA, SCCA, Adeno-squamous, ADCA *in-situ* (AIS, formerly BAC) or other.

### Tissue preparation

Tumour and non-adjacent lung tissues were received within 2 h after resection and immediately processed for flow cytometry analysis. The enzyme cocktail and time of tissue digestion were developed based on the findings of Grange *et al*.^37^ Tissue digestion methods were optimized using a training set of *N*=6 NSCLC specimens, with the primary goal to yield an immune cell rich digest with high cellular viability from both lung tissue and lung tumour tissue. Tissue was mechanically dissociated and subsequently digested in RPMI-1640 supplemented with 80 U ml^−1^ DNase I, 300 U ml^−1^ collagenase I and 60 U ml^−1^ hyaluronidase at 37 °C for 30 min. Enzymatic digests were&#8232;inactivated with FBS and single-cell suspension was passed through 70- and 40-μm cell strainers. After RBC lysis, cells were washed and resuspended in staining buffer (1 × PBS/2% FBS). Total and trypan blue-negative (viable) cells were counted with an automated cell counter (BioRad). Representative images of propidium iodide overlay and measurements of viability are provided in Supplementary Fig. 3. Backgating was used to demonstrate that all of the major immune cell types were isolated from both lung tissue and lung tumour tissue following digestion. Aliquots of single cell suspension were retained for DNA isolation.

### Flow cytometry

Flow cytometry staining was performed immediately after single-cell suspension was obtained and staining was performed in four separate panels. Fresh tumour and non-adjacent lung single cell suspensions were pre-incubated with Fc receptor blocking solution (Biolegend) to reduce non-specific binding and subsequently stained according to the specific protocols.

*Lymphocyte surface panel*. Cells were stained with fluorochrome-conjugated anti-human antibodies against CD45, CD3, CD4, CD8, CD19, CD20, CD56 and γδTCR (antibody and clone details, see Supplementary Table 2) for 30 min on ice, washed and stained with fixable viability dye (FVD, eBioscience), before fixation (IC fixation buffer, eBioscience).

*Lymphocyte function panel*. Cells were stained with fluorochrome-conjugated anti-human antibodies against CD45, CD3, CD4, CD8, CCR7, CD45RA, CD69, PD1 and TIM3 for 30 min on ice, washed and stained with FVD before fixation (IC fixation buffer).

*Myeloid surface panel*. Cells were stained with fluorochrome-conjugated anti-human antibodies against CD45, CD14, CD33, CD66b, EpCAM and PDL1 for 30 min on ice, washed and stained with FVD before fixation/permeabilization using BD Cytofix/Cytoperm kit (required for CD68 staining). CD68 staining was performed in perm/wash buffer at 4 °C overnight.

*Intracellular cytokine panel*. Cells were cultured in RPMI-1640 with 10% FBS, 1% Pen/Strep, 50 ng ml^−1^ PMA, 1 μg ml^−1^ ionomycin for 5 h at 37 °C with 5% CO_2_ (ref. 38). 1.5 μl ml^−1^ GolgiStop (monensin) was added for the last 2 h. Following stimulation, cells were washed, resuspended and stained with FVD. Subsequently, cells were fixed and permeabilized using a BD Cytofix/Cytoperm kit and stained O/N at 4 °C with fluorochrome-conjugated antibodies against CD45, CD3, CD4, CD8, CD25, CD127, γδTCR, IFNγ, IL-17A and IL-22.

The supplementary MDSC panel (*N*=8) was performed in similar fashion to the myeloid surface panel, above. Cells were stained with fluorochrome-conjugated anti-human antibodies against CD45, CD3, CD8, CD11b, CD14, CD15, CD33, CD66b and HLA-DR for 30 min on ice, washed and stained with FVD before fixation/permeabilization using BD Cytofix/Cytoperm kit (required for CD68 staining). CD68 staining was performed in perm/wash buffer at 4 °C overnight. In addition, sucrose density centrifugation was performed on blood specimens from this cohort as per the manufacturer's instructions (Histopaque-1077, Sigma). Cytospin preparations of the low-density fractions were used to tabulate the % of neutrophils (at least 200 total cells counted per slide).

At least 50,000 live events were collected per sample (BD LSR II Cytometer). Compensation was performed using single stains (Supplementary Fig. 1). Cutoffs for background fluorescence were based on the ‘fluorescence minus one' strategy^39^ (Supplementary Fig. 2). Briefly, each antibody within a given panel (except the antibody of interest) was utilized to identify background staining. For cytokine panel gating, unstimulated control (no PMA/ionomycin stimulation) was used^38^. Data were analysed using FloJo software (TreeStar). Gating for each sample is based on SSC-Height versus SSC-Width and FSC-Height versus FSC-Width plot to eliminate aggregates. FVD staining was used to identify and eliminate dead cells that were assessed using contour plots (Supplementary Fig. 3). Propidium iodide overlay was used to validate cellular viability in the training set (Supplementary Fig. 3). Cellular definitions were based upon recently published guidelines unless designated otherwise in the text^26^,^40^.

### Immunohistochemistry (IHC)

Formalin-fixed and paraffin-embedded human lung adenocarcinoma (*N*=37) and squamous cell carcinoma (*N*=9) cases (taken from the *N*=73 cohort) were obtained through NWBio (IRB protocol #6663). IHC staining was performed on the Leica Bond Automated Immunostainer. Sections were deparaffinized in Leica Bond Dewax Solution and rehydrated through 100% ETOH. After antigen retrieval (Citrate, Lieca Bond Epitope Retrieval Solution 1) at 100 °C for 20 min and blocking endogenous peroxidase activity with 3.0% H_2_O_2_ for 5 min and blocking with 10% Normal Goat Serum in TBS for 20 min the sections were incubated with primary antibody (CD45 LCA, clone 2B11+PD7/26, 1:600, DAKO) or matching IgG control, both in Leica Bond Primary Antibody Diluent for 30 min at room temperature. Subsequently, sections were incubated with anti-mouse poly-HRP polymer secondary detection for 8 min at room temperature, followed by incubation with Leica Bond Mixed Refine DAB substrate detection for 10 min at room temperature. After washing with diH_2_O the sections were counter stained with Hematoxylin solution (Leica Bond Refine Kit) dehydrated with 100% ETOH, cleared in Xylene and mounted with synthetic resin mounting medium. The CD45 stained slides were scanned on an Aperio AT Turbo slide scanner (Leica). Images were analysed using the HALO 2.0 Area Quantification algorithm (Indica Labs), a whole-slide imaging data analysis software program that measures and reports individual cell data that is represented as the percentage of positive cells per mm^2^ tissue^41^.

A subset of these cases (*N*=6L-ADCA and N=6L-SCCA) were stained with CD66b antibody (CD66b, clone G10F5, 1:250, BD) and processed in the same way as described for CD45, above.

### TCR sequencing

DNA was isolated from frozen single cell suspensions from corresponding patient samples using QIAamp DNA kit (Qiagen) per manufacturer's instructions. TCR-β immunosequencing was performed on the DNA from 60 NSCLC specimens and 34 non-adjacent lung tissue specimens with corresponding flow cytometry data by Adaptive Biotechnologies (Seattle, WA, USA) as previously described^16^,^42^. Briefly, input DNA was amplified in a two-step multiplex PCR in which the first PCR amplified the CDR3 region of T-cell genomes and the second PCR added adaptor sequences compatible with Illumina(R) next-generation sequencing (NGS) platform. Sequencing was performed using Illumina's NGS platform and data analysed through Adaptive Biotechnologies immunoSEQ Analyzer. The data were expressed as the Top 10 Clones (% of total T-cell templates originating from the 10 most frequent sequences) or productive (amino acid sequence) clonality score^15^,^43^,^44^. The clonality score quantifies the extent of mono- or oligoclonal expansion by measuring the frequency of clones within the distribution (it roughly distills the distribution of T cells down to one number that is nearly invariant with the size of distribution) and is based on the following mathematical equations:


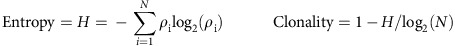


Clonality values range from 0 to 1, with a value of 1 representing a monoclonal population. TCR-β sequences only present in tumour but not in non-adjacent lung tissue (identified using log_10_ scatter plots) were defined as TAC.

### Generation of tumour-infiltrating lymphocyte (TIL) populations

TIL populations were generated from supplementary cohort #1 of *N*=10L-ADCA specimens for which paired (lung tissue and lung tumour tissue) TCR-β sequencing was also generated. We utilized a protocol previously optimized to generate and test the tumour reactivity of TIL from melanoma specimens^45^. Briefly, 6–12 fragments of L-ADCA tissue (2 × 2 × 2 mm) were cultured in 24-well plates in T-cell media (RPMI 1640, 10% FCS, 10 mM HEPES, 1 × Pen/Strep, 50 μg ml^−1^ gentamicin, 1 × 2-mercaptoethanol) in the presence of interleukin-2 (IL-2, 6,000 U ml^−1^) for 35 days. TILs were passaged when confluent. Following the conclusion of the 35-day expansion protocol, an aliquot of the TIL (50,000 cells) was co-cultured with autologous tumour cells (100,000 cells). The supernatant was subjected to IFNγ ELISA analysis following overnight culture (Biolegend). TIL only and tumour only specimens served as the controls. A fivefold increase in IFNγ release was considered a positive result for tumour reactivity.

### Statistics

Flow cytometry data were reported as cell composition, expressed as % of live cells, % of CD45^+^ cells, % of CD4^+^ cells or % of parent population. For functional markers, such as PD1, TIM3, PDL1 and so on, the results were reported and analysed as Median Fluorescence Intensity (MFI), which accounts for total expression of surface marker. Student's *t*-tests were used to compare results between two groups. These methods were used to determine differences between NSCLC and non-adjacent lung tissue or between L-ADCA and L-SCCA. ANOVA was used to assess differences between tumour stages. Linear regressions were used to study the relationship between two variables in this study. Scatter plots, regression lines and statistical analysis was performed using GraphPad Prism7 and R software ( http://www.r-project.org/). A *P* value of <0.05 was considered statistically significant.

### Data availability

The TCR sequencing data have been deposited in the ImmuneACCESS database under the accession code DOI 10.21417/B7B88G (URL: http://doi.org/10.21417/B7B88G). We declare that all the other data supporting the findings of this study are available within the article and its Supplementary Information Files and from the corresponding author upon reasonable request.

## Additional information

**How to cite this article:** Kargl, J. *et al*. Neutrophils dominate the immune cell composition in non-small cell lung cancer. *Nat. Commun.* **8,** 14381 doi: 10.1038/ncomms14381 (2017).

**Publisher's note:** Springer Nature remains neutral with regard to jurisdictional claims in published maps and institutional affiliations.

## Supplementary Material

Supplementary Information Supplementary Figures and Supplementary Tables
