# Supplementary material for: Neutrophils dominate the immune cell composition in non-small cell lung cancer
Source: Nat Commun. 2017 Feb 1;8:14381. doi: 10.1038/ncomms14381 (PMC5296654; doi:10.1038/ncomms14381)
Supplement: Supplementary Information — Supplementary Figures and Supplementary Tables [file ncomms14381-s1.pdf]

Supplementary Table 1: Patient Demographics

Factor	All NSCLC	Lung ADCA	Lung SCCA	Other
N	73	54 (74%)	15 (21%)	4 (5%)
**Age**	67.3	66.5	69.1	71.8
**% Male**	49	43	67	75
**Race**				
**White**	64 (88)	47 (87)	13 (86)	4 (100)
**Asian**	6 (8)	5 (9)	1(7)	0
**Other**	3 (4)	2 (4)	1 (7)	0
**Smoking**				
**Current**	20 (27)	15 (28)	5 (33)	0
**Former**	45 (62)	33 (61)	10 (67)	2 (50)
**Never**	8 (11)	6 (11)	0	2 (50)
**Pack Years**	29.9	27.9	41.1	13.8
**Size (cm)**	2.78	2.69	3.06	2.93
**Stage**				
**1A**	25 (34)	19 (35)	6 (40)	0
**1B**	23 (32)	18 (33)	3 (20)	2 (50)
**2A**	10 (14)	7 (13)	2 (13)	1 (25)
**2B**	4 (5)	2 (4)	1 (7)	1 (25)
**3A**	10 (14)	7 (13)	3 (20)	0
**4**	1 (1)	1 (2)	0	0

Supplementary Table 2: Immune Cell Markers

Antigen	Label	Clone	ul/test	Company	Catalog #
**CD14**	BUV395	MφP9	5	BD	563561
**CD16**	BV605	3G8	5	BD	563172
**CD3**	PE-CF594	UCHT1	5	BD	562280
**CD4**	BUV395	SK3	5	BD	563550
**IL17A**	PE	N49-653	20	BD	560486
**IFNγ**	FITC	B27	1	BD	554700
**CD127**	BV786	HIL-7R-M21	5	BD	563324
**CD19**	BUV737	SJ25C1	5	BD	564303
**CD15**	BV786	HI98	5	BD	563838
**HLA-DR**	BUV661	G46-6	5	BD	565073
**CD4**	BV605	OKT4	5	Biolegend	317438
**CD45**	AlexaFluor700	HI30	1	Biolegend	304024
**CD66b**	PE	G10F5	5	Biolegend	305106
**CD68**	APC	Y1/82A	5	Biolegend	333810
**CD274 (PDL1)**	PE-Cy7	29E.2A3	5	Biolegend	329718
**CD33**	BV421	WM53	5	Biolegend	303416
**CD4**	BV605	RPA-T4	5	Biolegend	317438
**CD25**	BV421	BC96	5	Biolegend	302630
**TCRγδ**	APC	B1.1	5	Biolegend	331212
**CD8**	BV510	RPA-T8	5	Biolegend	301048
**CD8**	FITC	RPA-T8	2	Biolegend	301006
**CD56**	PE	HCD56	5	Biolegend	318306
**CD20**	BV421	2H7	5	Biolegend	302330
**NKG2D**	PE-Cy7	1D11	5	Biolegend	320812
**CCR7**	PE-Cy7	G043H7	5	Biolegend	353226
**CD45RA**	APC	HI100	5	Biolegend	304112
**CD69**	BV605	FN50	5	Biolegend	310938
**PD1**	BV421	EH12.2H7	5	Biolegend	329920
**CD326 (EpCAM)**	FITC	9C4	5	Biolegend	324204
**CD11b**	BV650	ICRF44	5	Biolegend	301336
**IL22**	PE-Cy7	22URTI	5	eBioscience	25-7229-42
**CD19**	PerCPCy5.5	HIB19	5	eBioscience	45-0199-42
**FVD**	APC-ef780	-	0.2	eBioscience	65-0865-14
**TIM3**	PE	344823	5	R&D Systems	FAB2365P

Supplementary Table 3: Unpaired t-tests for lung vs. tumor and L-ADCA vs. L-SCCA

% of	Cell population	Lung vs. Tumor	ADCA vs SCCA
**% live**	CD45	0.82241	0.07023
**% live**	EpCAM	0.01065	0.92376
**% CD45**	CD3	0.00000	0.99500
**% CD45**	CD4	0.00001	0.24897
**% CD45**	CD8	0.00016	0.10618
**% CD45**	Tgd	0.65118	0.92503
**% CD45**	NK	0.00025	0.85798
**% CD45**	NKT	0.00991	0.34630
**% CD45**	Bcells	0.00000	0.56647
**% CD45**	Mac	0.00000	0.01975
**% CD45**	Mono	0.68578	0.71318
**% CD45**	CD14-CD33+	0.87744	0.27477
**% CD45**	PMN	0.37325	0.03282
**% gdT**	Tgd IL17A	0.00407	0.85899
**% gdT**	Tgd IFNg	0.85221	0.85580
**% gdT**	Tgd IL22	0.00849	0.13732
**% CD4**	CD4+ IL17A	0.05864	0.03528
**% CD4**	CD4+IL22	0.42312	0.31931
**% CD4**	CD4+IL17A+IL22+	0.01718	0.31057
**% CD4**	CD4+IFNg	0.00035	0.04707
**% CD4**	Treg	<0.00001	0.00006
**% CD4**	CD4EM	0.24833	0.20101
**% CD4**	CD4CM	0.16730	0.73166
**% CD4**	CD4naive	0.00543	0.41214
**% CD4**	CD4EMRA	0.00379	0.47215
**% CD4**	CD4+ CD69	0.60107	0.07469
**% CD4**	CD4+ PD1	0.00000	0.00253
**% CD4**	CD4+ TIM3	0.00016	0.11155
**% CD4**	CD4+ PD1-TIM3-	0.00000	0.02700
**% CD4**	CD4+ PD1+TIM3-	0.00012	0.01946
**% CD4**	CD4+ PD1+TIM3+	0.00000	0.44402
**% CD4**	CD4+ PD1-TIM3+	0.81601	0.25677
**% CD8**	CD8+IFNg	0.33900	0.98888
**% CD8**	CD8EM	0.00000	0.01226
**% CD8**	CD8CM	0.07400	0.86100
**% CD8**	CD8naive	0.21348	0.70970
**% CD8**	CD8EMRA	0.00000	0.00643
**% CD8**	CD8+ CD69	0.18627	0.12426
**% CD8**	CD8 PD1	0.00000	0.01085
**% CD8**	CD8+ TIM3	0.00000	0.66851
**% CD8**	CD8+ PD1-TIM3-	0.00000	0.01559
**% CD8**	CD8+ PD1+TIM3-	0.00377	0.01881
**% CD8**	CD8+ PD1+TIM3+	0.00000	0.47425
**% CD8**	CD8+ PD1-TIM3+	0.32673	0.58201
**MFI**	CD4+ CD69	0.63653	0.24495
**MFI**	CD4+ PD1	0.00006	0.00952
**MFI**	CD4+TIM3	0.00184	0.42447
**MFI**	CD8+CD69	0.00030	0.19333
**MFI**	CD8+PD1	0.00000	0.02811
**MFI**	CD8+ TIM3	0.00000	0.90053
**MFI**	NKT NKG2D	0.00047	0.72978
**MFI**	NK NKG2D	0.44371	0.39137

**MFI**	Tgd NKG2D	0.00899	0.27486
**MFI**	total PD-L1	0.02706	0.16392
**MFI**	PMN PD-L1	0.00346	0.04823
**MFI**	Mac PD-L1	0.13175	0.67825
**MFI**	Mono PD-L1	0.01229	0.01020
**MFI**	CD45- PD-L1	0.04190	0.09140
**MFI**	EpCAM PD-L1	0.00202	0.01953

Supplementary Table 4: Unpaired t-tests for lung vs. tumor and L-ADCA vs. L-SCCA (all data expressed as % of CD45)

**% of**	**Cell population**	**Lung vs Tumor**	**ADCA vs SCCA**
**% CD45**	CD3	0.00000	0.99500
**% CD45**	CD4	0.00001	0.24897
**% CD45**	CD8	0.00016	0.10618
**% CD45**	Tgd	0.65118	0.92503
**% CD45**	NK	0.00025	0.85798
**% CD45**	NKT	0.00991	0.34630
**% CD45**	Bcells	0.00000	0.56647
**% CD45**	Mac	0.00000	0.01975
**% CD45**	Mono	0.68578	0.71318
**% CD45**	CD14-CD33+	0.87744	0.27477
**% CD45**	PMN	0.37325	0.03282
**% CD45**	Tgd IL17	0.00007	0.63034
**% CD45**	Tgd IFNg	0.00018	0.39253
**% CD45**	Tgd IL22	0.00048	0.37731
**% CD45**	CD4+ IL17	0.00002	0.06999
**% CD45**	CD4+IL22	0.00019	0.15406
**% CD45**	CD4+IL17+IL22+	0.00025	0.16325
**% CD45**	CD4+IFNg	0.20615	0.05323
**% CD45**	Treg	<0.00001	0.14309
**% CD45**	CD4EM	0.00001	0.64308
**% CD45**	CD4CM	0.03315	0.67581
**% CD45**	CD4naive	0.47011	0.44593
**% CD45**	CD4EMRA	0.57728	0.59177
**% CD45**	CD4+ CD69	0.03388	0.12500
**% CD45**	CD4+ PD1	0.00000	0.22081
**% CD45**	CD4+ TIM3	0.00004	0.18678
**% CD45**	CD4+ PD1-TIM3-	0.42348	0.14813
**% CD45**	CD4+ PD1+TIM3-	0.00000	0.54021
**% CD45**	CD4+ PD1+TIM3+	0.00000	0.70350
**% CD45**	CD4+ PD1-TIM3+	0.09197	0.34784
**% CD45**	CD8+IFNg	0.00510	0.00699
**% CD45**	CD8EM	0.00000	0.00737
**% CD45**	CD8CM	0.02628	0.40778
**% CD45**	CD8naive	>0.99999	0.21574
**% CD45**	CD8EMRA	0.13812	0.37237
**% CD45**	CD8+ CD69	0.00503	0.58720
**% CD45**	CD8 PD1	0.00000	0.00388
**% CD45**	CD8+ TIM3	0.00001	0.36818
**% CD45**	CD8+ PD1-TIM3-	0.48928	0.97415
**% CD45**	CD8+ PD1+TIM3-	0.00000	0.00079
**% CD45**	CD8+ PD1+TIM3+	0.00000	0.18855

**% CD45**	CD8+ PD1-TIM3+	0.01149	0.94173

Supplementary Table 5: Unpaired t-tests for lung vs. tumor and L-ADCA vs. L-SCCA (all data expressed as % live cells)

% live	Cell population	Lung vs. Tumor	ADCA vs SCCA
**% live**	CD45	0.82241	0.07023
**% live**	EpCAM	0.01065	0.92376
**% live**	CD3	0.00001	0.6991
**% live**	CD4	0.00005	0.50106
**% live**	CD8	0.00036	0.08976
**% live**	Tgd	0.77885	0.78054
**% live**	NK	0.00016	0.64302
**% live**	NKT	0.01015	0.17846
**% live**	Bcells	0.00000	0.40496
**% live**	Mac	0.00000	0.04052
**% live**	Mono	0.51530	0.95403
**% live**	CD14-CD33+	0.84399	0.28161
**% live**	PMN	0.43756	0.00984
**% live**	Tgd IL17	0.00008	0.74648
**% live**	Tgd IFNg	0.00046	0.60967
**% live**	Tgd IL22	0.00040	0.31897
**% live**	CD4+ IL17	0.00002	0.09807
**% live**	CD4+IL22	0.00022	0.18593
**% live**	CD4+IL17+IL22+	0.00067	0.19046
**% live**	CD4+IFNg	0.28961	0.10198
**% live**	Treg	0.00000	0.0987
**% live**	CD4EM	0.00010	0.77226
**% live**	CD4CM	0.02255	0.66526
**% live**	CD4naive	0.54511	0.42745
**% live**	CD4EMRA	0.63671	0.61308
**% live**	CD4+ CD69	0.05484	0.17963
**% live**	CD4+ PD1	0.00000	0.20101
**% live**	CD4+ TIM3	0.00010	0.1636
**% live**	CD4+ PD1-TIM3-	0.57364	0.16856
**% live**	CD4+ PD1+TIM3-	0.00000	0.48527
**% live**	CD4+ PD1+TIM3+	0.00000	0.56738
**% live**	CD4+ PD1-TIM3+	0.06542	0.38413
**% live**	CD8+IFNg	0.01215	0.01252
**% live**	CD8EM	0.00000	0.00311
**% live**	CD8CM	0.02532	0.3698

**% live**	CD8naive	>0.999999	0.10423
**% live**	CD8EMRA	0.10679	0.70531
**% live**	CD8+ CD69	0.00701	0.61274
**% live**	CD8 PD1	0.00000	0.00753
**% live**	CD8+ TIM3	0.00001	0.43539
**% live**	CD8+ PD1-TIM3-	0.34422	0.87999
**% live**	CD8+ PD1+TIM3-	0.00002	0.00285
**% live**	CD8+ PD1+TIM3+	0.00000	0.21704
**% live**	CD8+ PD1-TIM3+	0.01421	0.9417

Supplementary Table 6: Linear regression associating cell composition and MFI with control, tumor size, and pack years (PY) smoking

Population	Control coefficients	Size coefficients	PY coefficients	Control p-value	Size p-value	PY p-value
**CD45**	0.3312 ±0.09231	1.759 ± 1.115	0.07386 ± 0.07148	0.0006	0.1192	0.3050
**CD3**	0.2014 ± 0.09119	-1.737 ± 1.245	0.1884 ± 0.09356	0.0307	0.1675	0.0481
**CD4**	0.3197 ± 0.1043	-1.223 ± 0.7578	0.04572 ± 0.05098	0.0031	0.1113	0.3729
**CD8**	0.1607 ± 0.07470	-0.6643 ± 0.7019	0.09401 ± 0.04513	0.0350	0.3472	0.0410
**γδ T**	0.8634 ± 0.1752	0.03111 ± 0.07434	0.006777 ± 0.004743	< 0.0001	0.6770	0.1576
**NK**	0.8407 ± 0.2065	-0.2598 ± 0.1545	0.002702 ± 0.01249	0.0001	0.0973	0.8294
**NK-T**	1.524 ± 0.2342	0.05241 ± 0.1289	0.01434 ± 0.009610	< 0.0001	0.6857	0.1404
**B**	0.003692 ± 0.02025	-0.06123 ± 0.3436	0.01480 ± 0.02668	0.8559	0.8591	0.5811
**Macrophage**	0.1795 ± 0.2236	-1.050 ± 0.8958	-0.09606 ± 0.05823	0.4249	0.2452	0.1039
**Monocyte**	0.1753 ± 0.06969	0.9495 ± 0.4745	-0.03354 ± 0.03239	0.0144	0.0498	0.3044
**$CD14^-CD33^+$**	0.3351 ± 0.1210	-0.8341 ± 1.164	-0.04670 ± 0.09277	0.0076	0.4767	0.6167
**Neutrophil**	0.08579 ± 0.1291	3.626 ± 0.8827	-0.001736 ± 0.06593	0.5086	0.0001	0.9791
**EpCAM**	0.1406 ± 0.1079	-1.378 ± 0.8848	0.02942 ± 0.06869	0.2013	0.1271	0.6707
**γδ T-IL17**	-38.84 ± 84.80	0.01561 ± 0.3021	-0.01855 ± 0.02365	0.6486	0.9589	0.4358
**γδ T-IFNγ**	0.4196 ± 0.1279	-1.886 ± 1.482	0.004953 ± 0.1179	0.0017	0.2081	0.9666
**γδ T-IL22**	0.03459 ± 0.03067	0.7857 ± 0.3331	0.01791 ± 0.02777	0.2645	0.0221	0.5219
**CD8-IFNγ**	0.1734 ± 0.1380	1.969 ± 2.918	-0.1019 ± 0.1107	0.2148	0.5215	0.3616
**Th17**	0.2303 ± 0.09434	0.004461 ± 0.2326	-0.009093 ± 0.01536	0.0174	0.9848	0.5560
**Th22**	0.3134 ± 0.1925	-0.1063 ± 0.1017	0.003830 ± 0.008164	0.1093	0.3006	0.6410
**$CD4^+IL17A^+IL22^+$**	0.2747 ± 0.06858	-0.03911 ± 0.06193	-4.025e-005 ± 0.0048	0.0002	0.5305	0.9934
**Th1**	0.6169 ± 0.1041	-3.256 ± 1.454	0.05526 ± 0.09883	< 0.0001	0.0286	0.5780
**Treg**	0.08373 ± 0.04710	1.305 ± 0.6229	0.08739 ± 0.04606	0.0802	0.0402	0.0623
**CD4EM**	0.8804 ± 0.1128	0.2945 ± 0.8164	-0.02053 ± 0.06708	< 0.0001	0.7201	0.7608
**CD4CM**	0.7811 ± 0.1126	-0.2779 ± 0.5985	-0.0009962 ± 0.0444	< 0.0001	0.6443	0.9822
**CD4Naive**	1.066 ± 0.1698	-0.04392 ± 0.09992	-0.006663 ± 0.00733	< 0.0001	0.6620	0.3675
**CD4EMRA**	1.007 ± 0.08827	0.7829 ± 0.7691	0.02836 ± 0.05752	< 0.0001	0.3133	0.6240
**CD8EM**	0.4510 ± 0.1445	1.688 ± 1.240	0.1416 ± 0.08856	0.0029	0.1790	0.1156
**CD8CM**	1.461 ± 0.1185	-0.4273 ± 0.3619	-0.01774 ± 0.02651	< 0.0001	0.2429	0.5061
**CD8Naive**	1.040 ± 0.1042	-0.1031 ± 0.09820	-0.01404 ± 0.006890	< 0.0001	0.2983	0.0464
**CD8EMRA**	0.5094 ± 0.1572	-1.268 ± 1.219	-0.1385 ± 0.08629	0.0021	0.3026	0.1141
**CD8CD69**	0.8705 ± 0.05154	-0.3617 ± 2.258	0.02389 ± 0.1777	< 0.0001	0.8733	0.8936
**CD8PD1**	0.6092 ± 0.1076	0.4014 ± 1.653	0.2259 ± 0.1254	< 0.0001	0.8091	0.0773
**CD8TIM3**	0.06222 ± 0.03224	0.8838 ± 1.039	0.1116 ± 0.08032	0.0589	0.3988	0.1704
**CD4CD69**	0.9283 ± 0.05027	-3.066 ± 2.062	-0.1545 ± 0.1621	< 0.0001	0.1432	0.3450
**CD4PD1**	0.5593 ± 0.09301	3.356 ± 1.639	0.2460 ± 0.1271	< 0.0001	0.0457	0.0583
**CD4TIM3**	0.2042 ± 0.04277	1.251 ± 0.6378	-0.02327 ± 0.05126	< 0.0001	0.0552	0.6517
**MFI**						
**NK-T NKG2D**	1.105 ± 0.1120	-6.791 ± 8.921	-0.3963 ± 0.7181	< 0.0001	0.4497	0.5832
**NK NKG2D**	0.9211 ± 0.1064	2.864 ± 5.806	-0.5888 ± 0.4585	< 0.0001	0.6237	0.2044
**γδ T NKG2D**	0.9805 ± 0.1651	-12.03 ± 6.655	-0.5203 ± 0.5428	< 0.0001	0.0763	0.3421
**CD8CD69**	0.8375 ± 0.08911	-18.63 ± 24.99	-1.500 ± 1.802	< 0.0001	0.4593	0.4087
**CD8PD1**	0.09831 ± 0.07068	42.81 ± 22.28	4.469 ± 1.666	0.1700	0.0598	0.0096
**CD8TIM3**	0.1390 ± 0.04129	3.801 ± 7.113	1.304 ± 0.5296	0.0014	0.5952	0.0170
**CD4CD69**	0.9715 ± 0.07771	-9.670 ± 15.65	-0.9069 ± 1.126	< 0.0001	0.5393	0.4243
**CD4PD1**	0.2020 ± 0.06215	95.53 ± 23.22	2.079 ± 2.049	0.0020	0.0001	0.3149
**CD4TIM3**	0.1984 ± 0.04289	9.819 ± 4.906	0.6699 ± 0.3852	< 0.0001	0.0504	0.0877
**PMN PDL1**	0.3927 ± 0.07647	-2.603 ± 9.524	0.03010 ± 0.8210	< 0.0001	0.7856	0.9709
**Mac PDL1**	0.6949 ± 0.1321	-31.74 ± 48.54	-0.1372 ± 4.016	< 0.0001	0.5159	0.9729
**Mono PDL1**	0.6166 ± 0.08614	4.838 ± 26.21	0.1803 ± 2.103	< 0.0001	0.8542	0.9320
**$CD45^-$ PDL1**	0.4836 ± 0.05650	7.593 ± 11.20	0.3806 ± 0.8961	< 0.0001	0.5003	0.6725
**EpCAM PDL1**	0.06115 ± 0.04264	-10.63 ± 14.34	1.630 ± 1.066	0.1588	0.4627	0.1334

Supplementary Table 7: Immune cell populations with significant differences by clinical stage (Two-Way ANOVA)

	Cell population	stage	NSCLC	ADCA
**MFI**	CD4+ PD1	1a vs. 3/4	<0.0001	<0.0001
**MFI**	total PD-L1	1a vs. 3/4	<0.0001	<0.0001
**MFI**	Mono PD-L1	1a vs. 3/4	<0.0001	0.0006
**MFI**	CD8+ PD1	1a vs. 3/4	ns	0.0013
**MFI**	Mac PD-L1	1a vs. 3/4	ns	0.0056

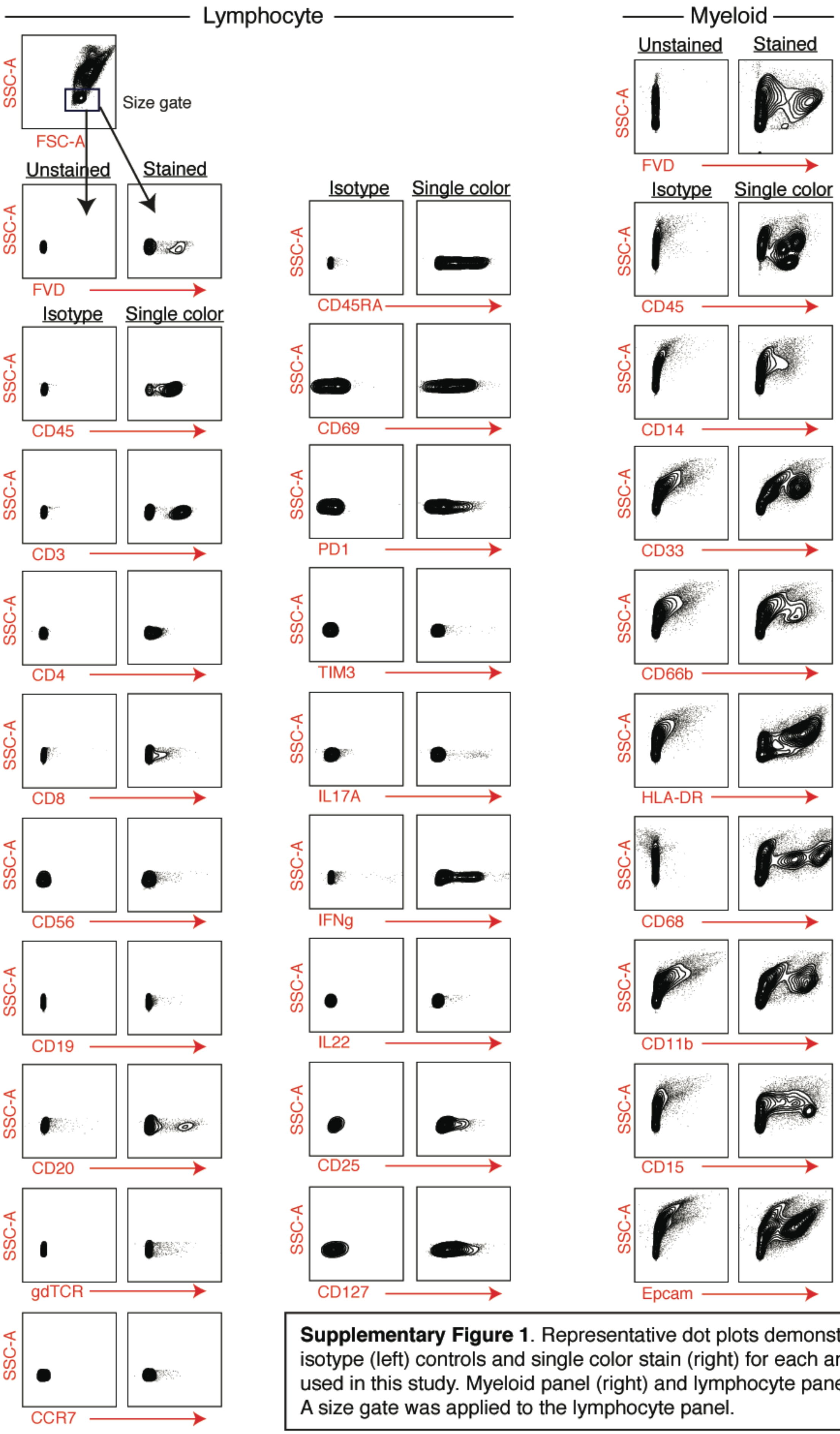

Supplementary Figure 1. Representative dot plots demonstrating isotype (left) controls and single color stain (right) for each antibody used in this study. Myeloid panel (right) and lymphocyte pane (left). A size gate was applied to the lymphocyte panel.

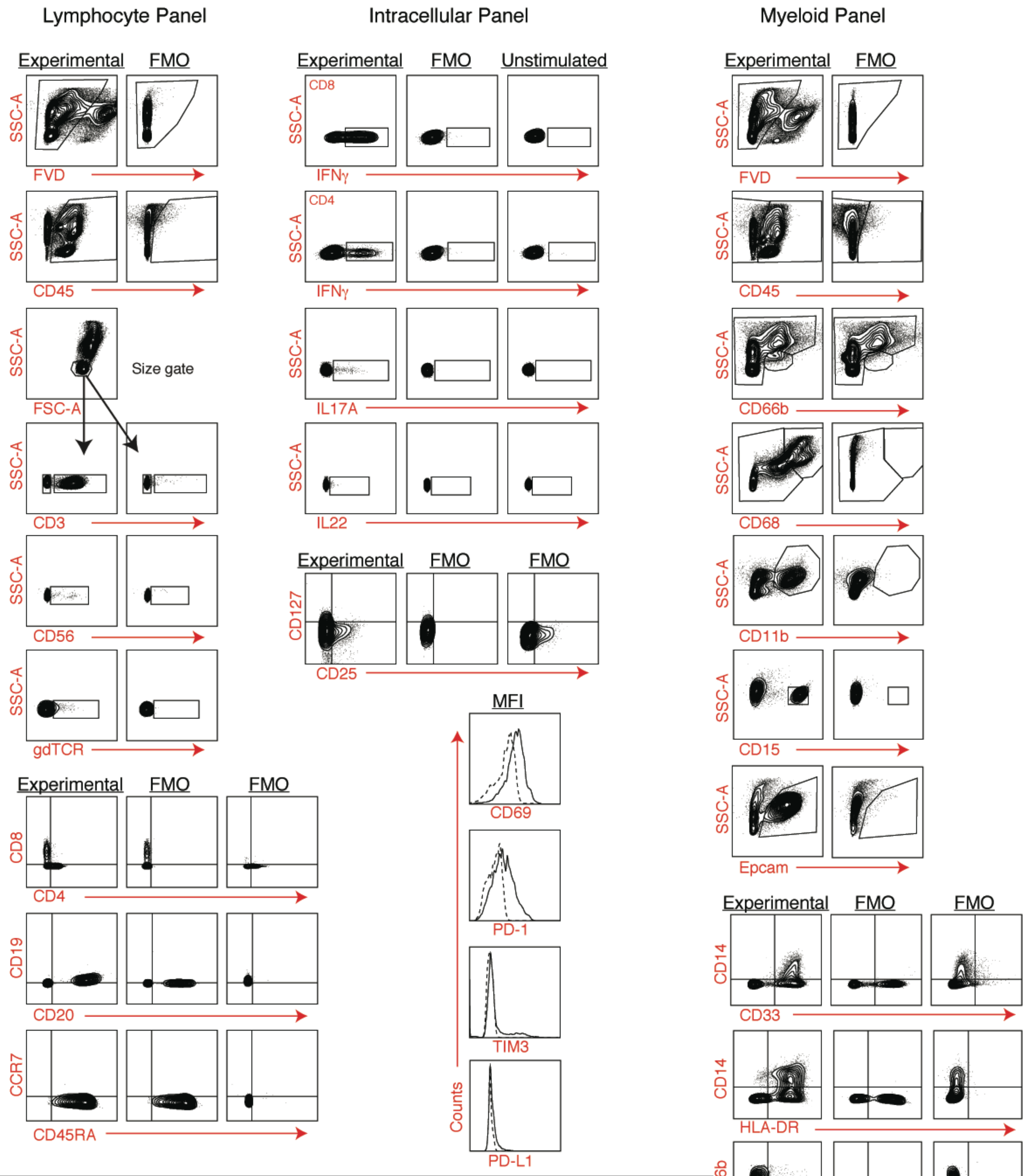

Supplementary Figure 2. Representative dot plots demonstrating gating controls. Experimental sample (multicolor stain, left), fluorescence minus one control (FMO, right) for lymphocyte panels, left; intacellular panel, middle; and myeloid panel, right; are displayed. The unstimulated conrol (only intracellular panel) was used for gating of cytokines (Il17, IFNγ, IL22). Median fluorescence intensity (MFI) was used for functional markers (CD69, PD1, TIM3, PDL1; FMO=dotted line, multicolor stain=black line; middle panel, bottom.

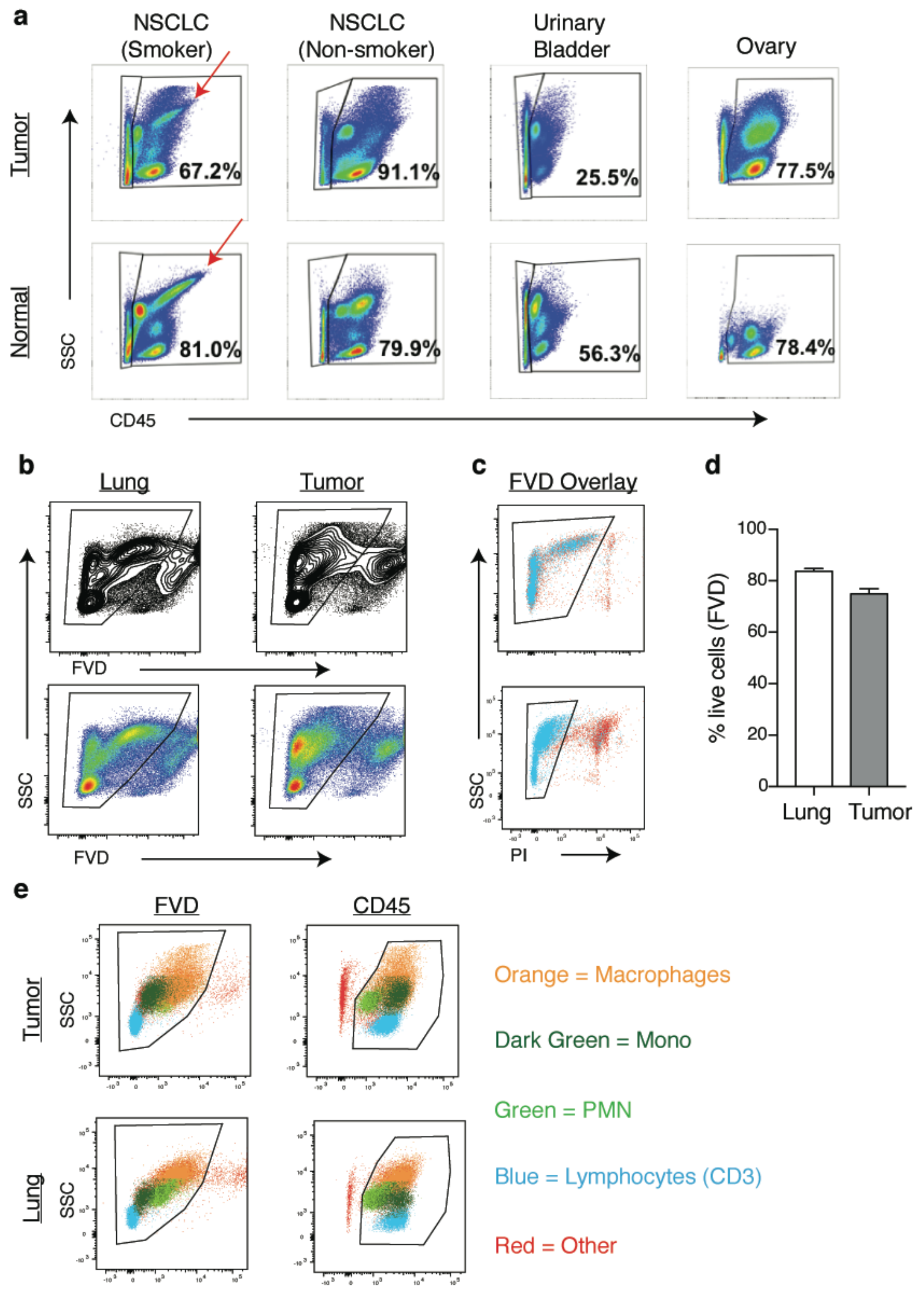

Supplementary Figure 3. a) CD45^{+} staining of tumor and matched normal tissue for representative cases from NSCLC (smoker), NSCLC (non-smoker), urinary bladder cancer, and ovarian carcinoma. Deptie identical staining protocols, the percentage of cells taing for CD45 viries by case and tissue. Red arrow depicts auto-fluorescent macrophages present in tumor and normal tissue from NSCLC smoker cases but not seen in other cases.
b) Representative dot plots for lung and tumor displaying fixable viability dy (FVD) staining as contour (top) and pseudocolor (bottom). Contour plots were used for garting on live cells (FVD negative). c) FVD and prodidium iodide (PI) co-staining were initally performed on lung and tumor single cell suspensions to examine differenct methods to discriminate live from dead cells (N=6). FVD and PI define similar live cell populations. FVD negative gated cells (blue) overlay on PI staining (red). FVD was used throughout the study to discrinimate live from dead cells. d) Tabulation of live cells (FVD negative) for lung and tumor specimens (N=73), P=NS. e) Major cell populations were backgated on FVD gate (left panels) and CD45 gate (right panels) to confirm live/dead gating (two representativelung and tumor specimens).

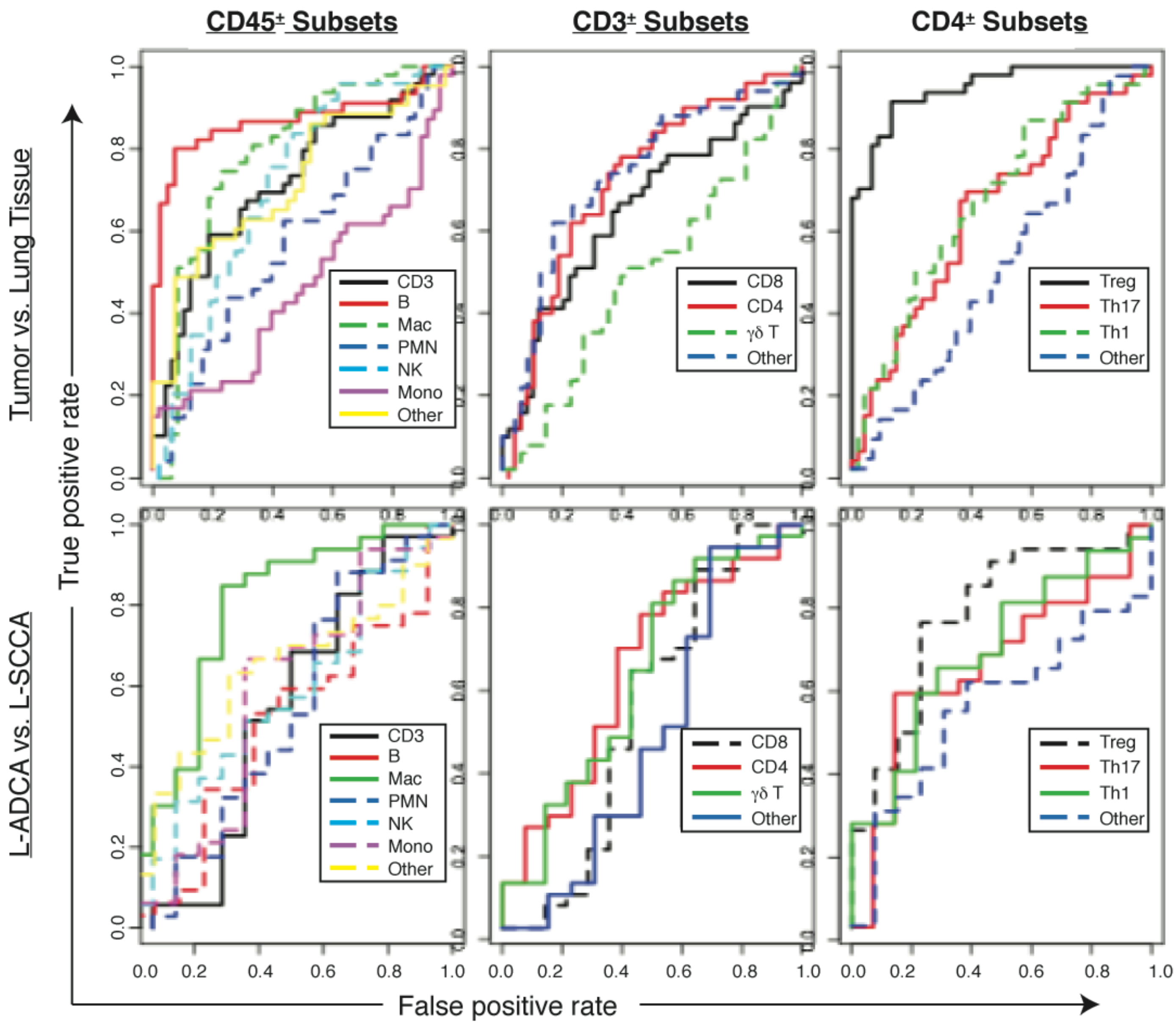

Supplementary Figure 4. Receiver Operating Characteristic (ROC) Curves showing discrimination ability of cell subset markers to differentiate tumor from normal lung tissue (top) and L-ADCA from L-SCCA (bottom). Solid lines indicate cell types that are more extreme in tumor vs. lung tissue (or L-ADCA vs. L-SCCA), and dotted lines indicate cell types that are more extreme in lung tissue vs. tumor (or L-SCCA vs. L-ADCA). The cell type marked “other” represents the difference between the total cell composition of the subset and 100% (i.e. the fraction otherwise not accounted for).

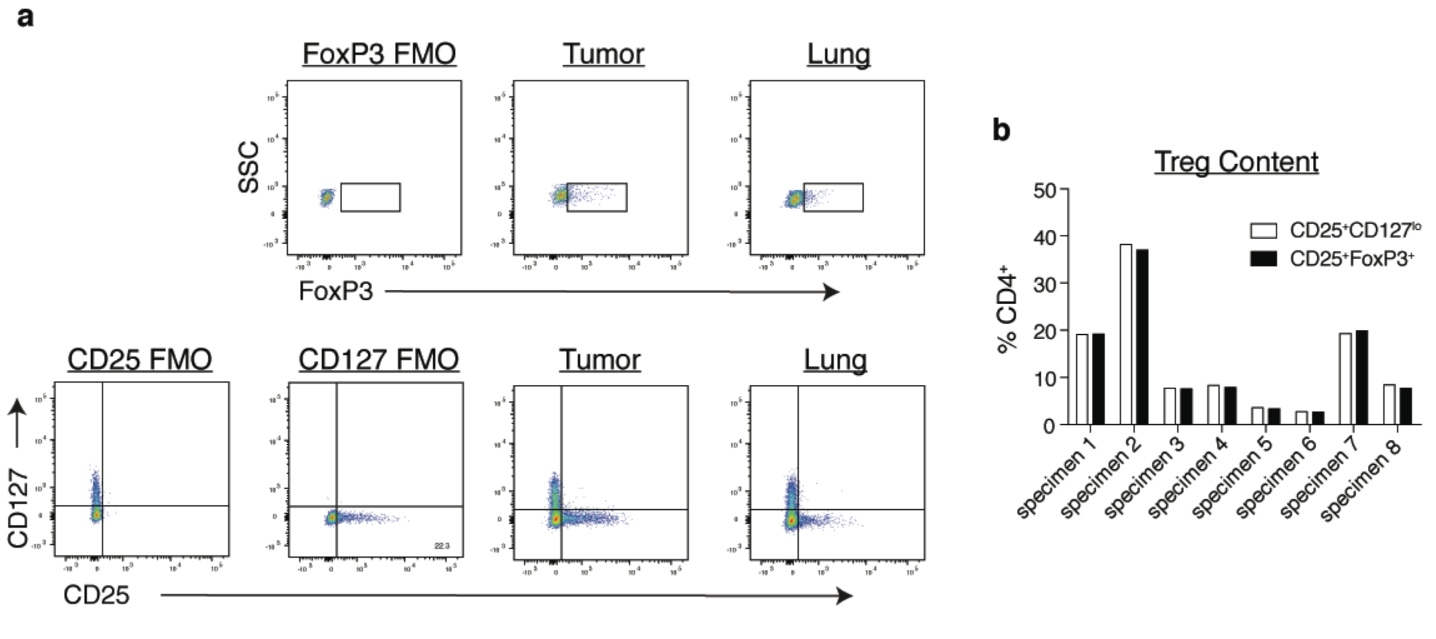

Supplementary Figure 5. The $CD4^+CD25^+FoxP3^+$ and $CD4^+CD25^+CD127^{lo}$ definitions of Tregs were compared on a set of N=8 NSCLC specimens. a) Representative gating of FoxP3 (top) and CD25/CD127 (bottom) depicting FMO controls (left) and tumor and lung (right). b) Tabulation of Tregs shows that both methods identified a similar number of cells as Tregs. The $CD25^+CD127^{lo}$ methods was used during the remainder of the study to quantify Tregs.

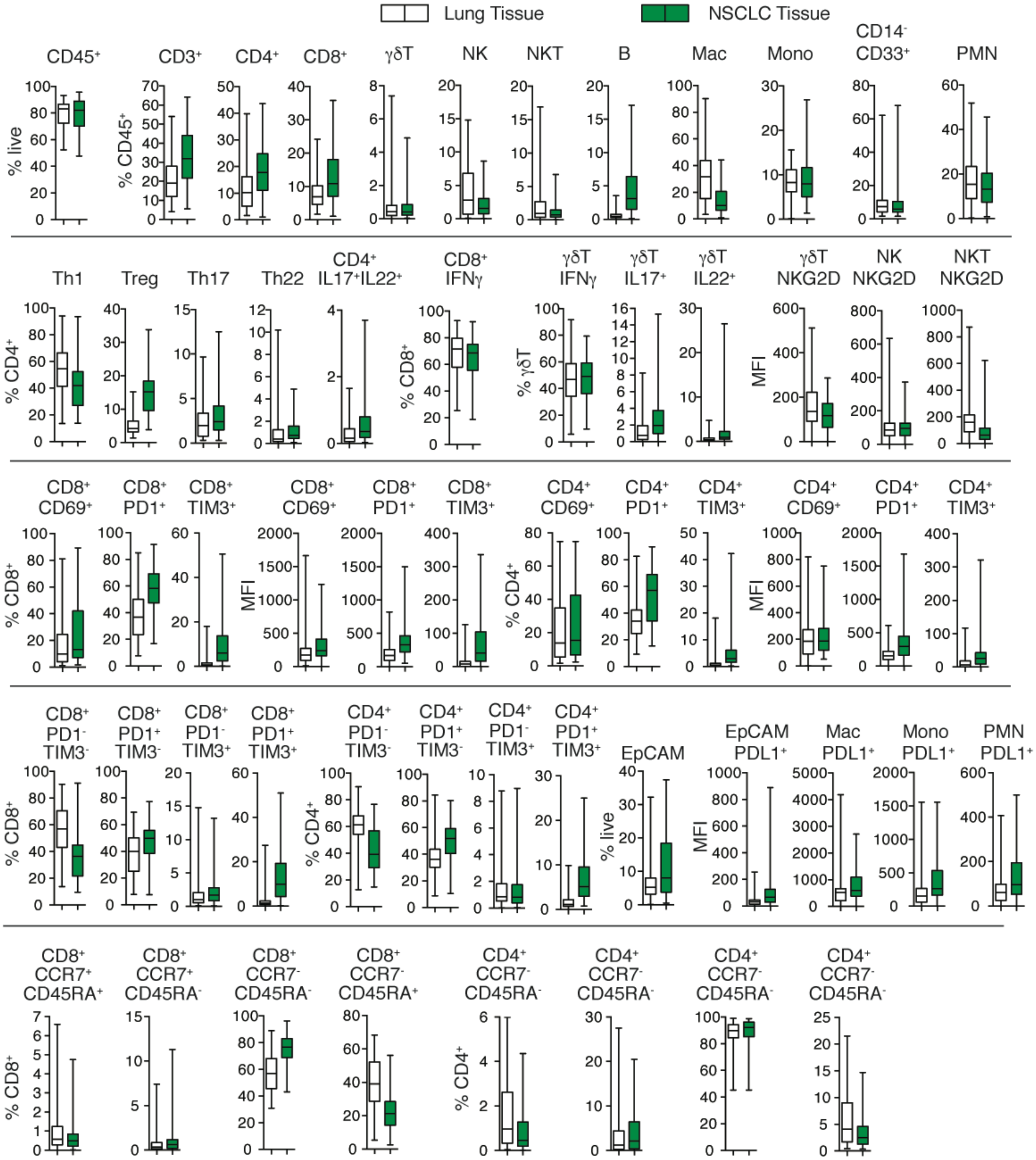

Supplementary Figure 6. Comparison of immune cell composition in lung tissue (white) and NSCLC tissue (green) for all cell populations analyzed. Data are presented as % live, %CD45+, % parent, or median fluorescence intensity (MFI), as indicated. Statistical analysis for each comparison is provided in Supplmentary Table 3.

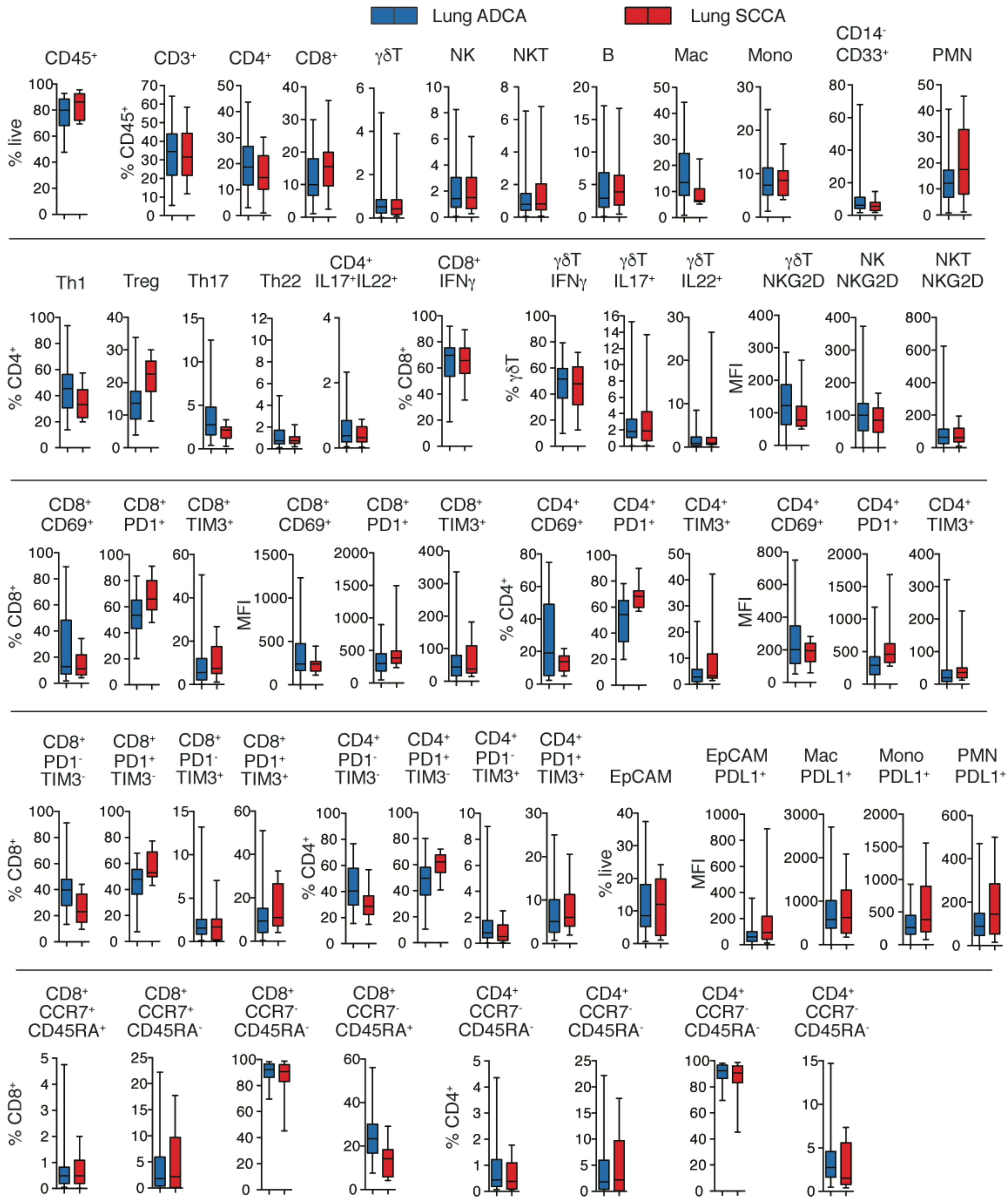

Supplementary Figure 7. Comparison of immune cell composition in L-ADCA (blue) and L-SCCA (red) specimens for all cell populations analyzed. Data are presented as % live, % CD45+, % parent or median fluorescence intensity (MFI), as indicated. Statistical analysis for each comparison is provided in Supplementary Table 3.

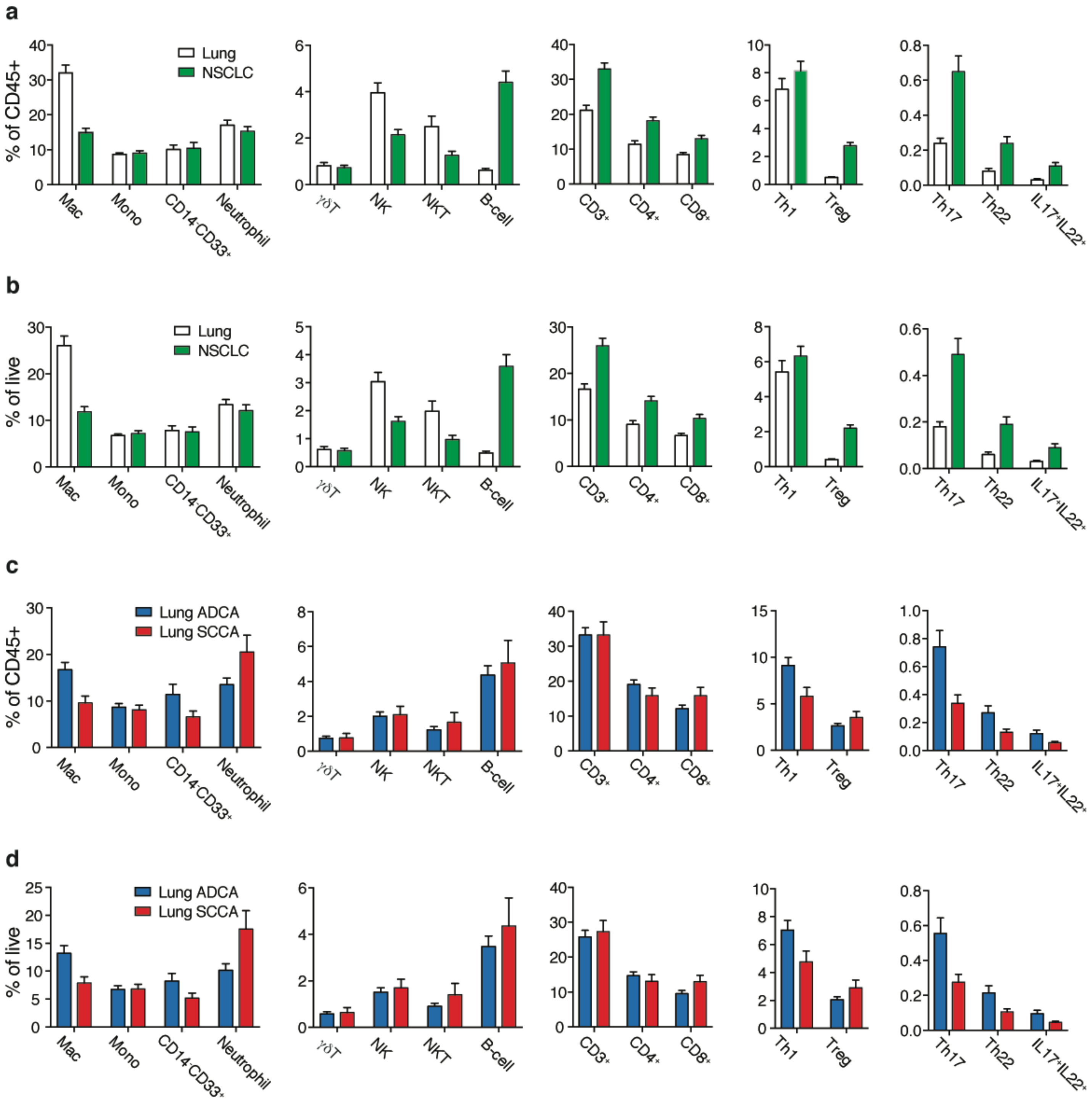

Supplementary Figure 8. Tabulation of immune cell content in non-adjacent lung tissue (white bars), NSCLC tissue (green bars), L-ADCA (blue bars) and L-SCCA (red bars) as measured by flow cytometry. Data are expressed as % of CD45⁺ cells (a and c) and as % of live cells (b and d).

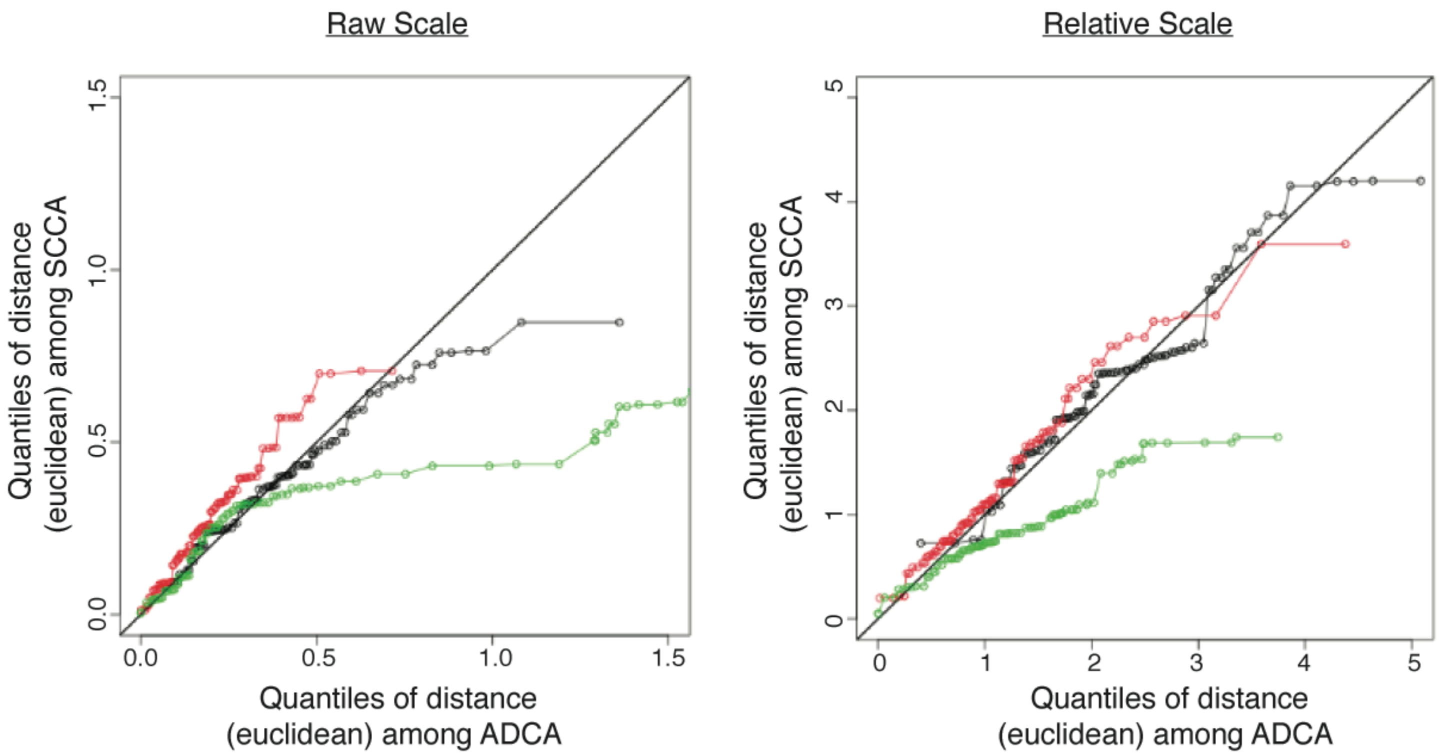

Supplementary Figure 9. Quantile plots comparing euclidean distance within the appropriate geometry for compositional data (see compositions package in R) of patient samples between ADCA (horizontal axis) and SCCA (vertical axis) for $CD45^+$ subsets (black), $CD3^+$ subsets (red), and $CD4^+$ subsets (green). The strong deviation of $CD4^+$ susets away from the line indicate its higher levels of heterogeneity compared to SCCA. On the raw scale, the increased heterogeneity appears to be driven by a small fraction of the cases. On the relative scale, the deviation can be seen to occur throughout the entire distribution.

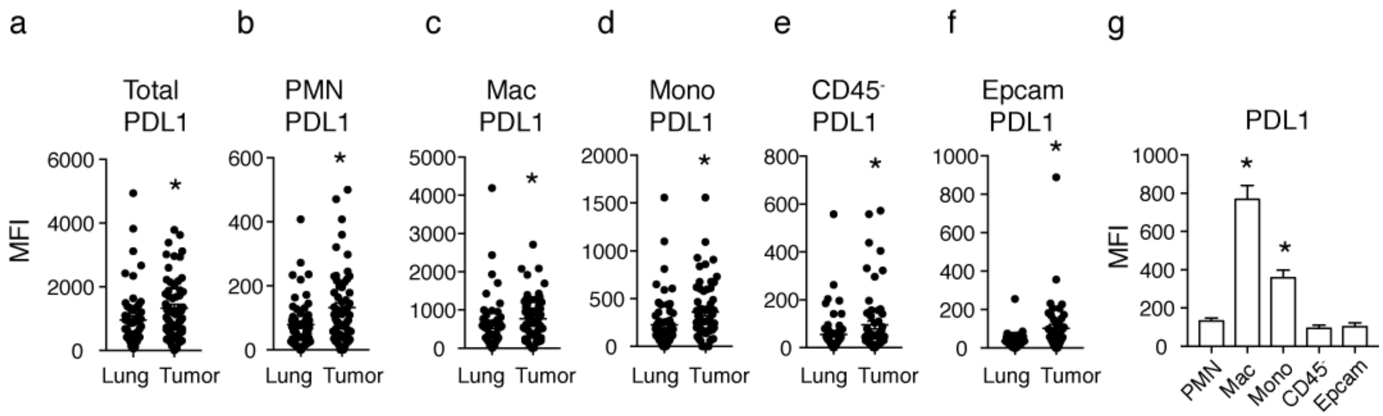

Supplementary Figure 10. PDL1 expression (MFI) from lung tissue and lung tumor for a) all cells, b) neutrophils, c) macrophages, d) monocytese) $CD45^-$ cells, and f)$Epcam^+$ cells as measured by flow cytometry. g) Tabulation of PDL1 expression by cell type. Bars +/- SEM. *P<0.05 (student's t-test).

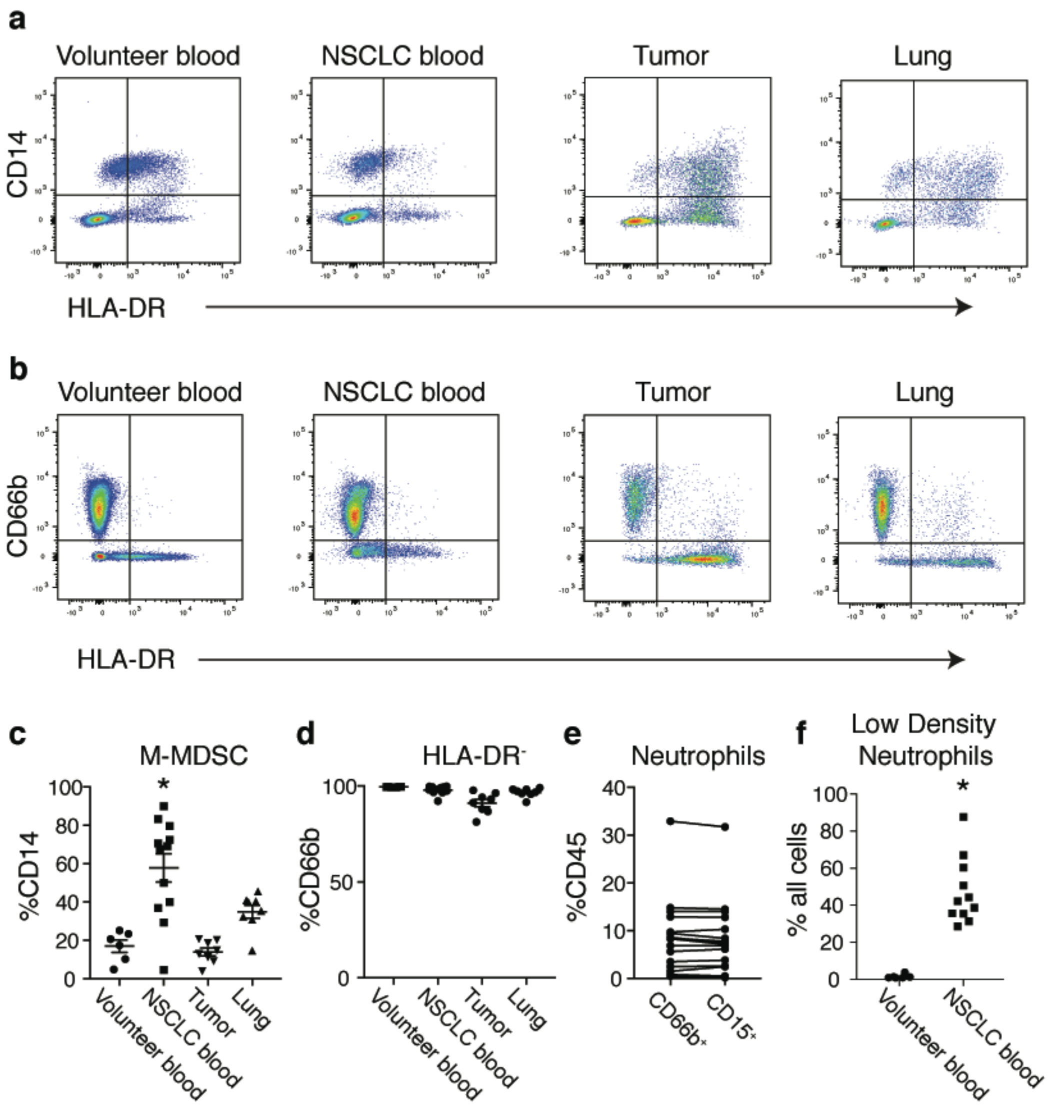

Supplementary Figure 11. Representative dot plots for myeloid derived suppressor cell (MDSC) gating. a) M-MDSC ($CD11b^+CD14^+HLA\text{-}DR^{lo}$) and b) the neutrophil lineage ($CD66b^+/HLA\text{-}DR^-$) from healthy volunteer donor blood (far left), NSCLC patient blood (left), tumor tissue (right) and lung tissue (far right). Tabulation of c) M-MDSC and d) $CD66b^+HLA\text{-}DR^-$ cells. e) CD66b and CD15 identify an equivalent number of cells as neutrophils (N=16). f) Tabulation of neutrophils located in the low density fraction following sucrose density gradient centrifugation for blood from healthy donor volunteers (N=6) and NSCLC patients (N=8). *P<0.05.

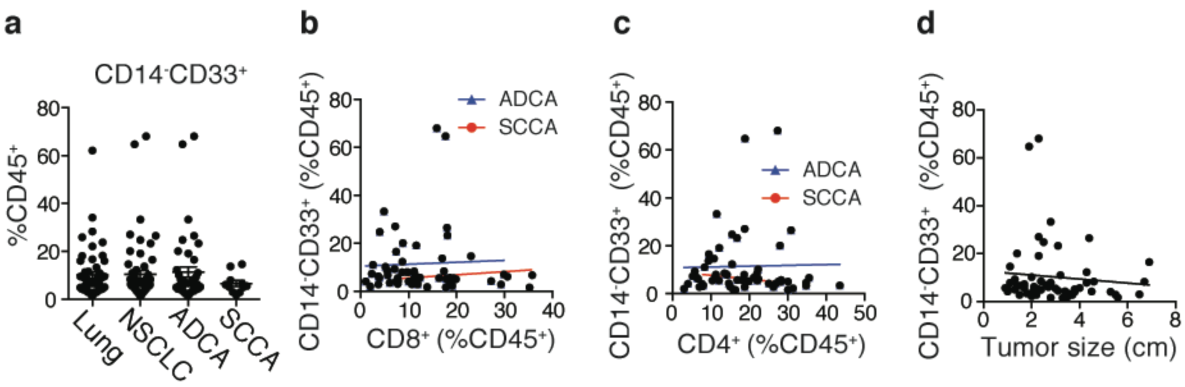

Supplementary Figure 12. a) Tabulation of CD14$^-$CD33$^+$ cells in lung tissue and tumor tissue. Linear correlation of CD14$^-$CD33$^+$ cells and b) CD8$^+$ cells, c) CD4$^+$ cells, and d) tumor tissue. P=NS for each regression.
